# Population mental health matters child health disparity: a national level analysis

**DOI:** 10.1186/s12889-022-14530-w

**Published:** 2022-12-17

**Authors:** Yao Yao, Yujie Cui, Yanfeng Zhang, Heng Li, Wu Zeng

**Affiliations:** 1 Shanghai Jiao Tong University School of Medicine, 227 South Chongqing Road, Huangpu District, Shanghai, 200025 P.R. China; 2 China Hospital Development Institute, Shanghai Jiao Tong University, Shanghai, China; 3 School of Economics and Management, Harbin Engineering University, 145, Nantong Street, Nangang District, Harbin, 150001 P.R. China; 4 Public Health Governance Research Center, East China University of Political Science and Law, 1575 Wandu Hang Road, Songjiang District, Shanghai, 200042 P.R. China; 5 Department of Global Health, School of Health, Georgetown University, 3700 Reservoir Rd NW, Washington, DC, USA

**Keywords:** Mortality rate under five, Disparity, Population mental health, Population mental health-gradient decomposition, Gender difference

## Abstract

**Background:**

The association between social distress and child health is important and attracts research interest. This study aims to examine the trend of inequality in the mortality rate for children under five (U5MR) over time and decompose the population mental health (PMH)-gradient in U5MR into different drivers at the national level.

**Methods:**

Data from 1990 to 2019 on the U5MR, PMH, and potential risk factors, such as socioeconomic status, environmental exposures at the national level, health behavior, basic water and sanitation services, urbanization, healthcare level, and HIV prevalence, were collected from online databases. We described the trend of U5MR and broke down U5MR based on the countries’ risk factor status and PMH. We constructed regression models and decomposed the drivers of change in U5MR disparity based on PMH-gradient.

**Results:**

The difference in U5MR between countries with different levels of air pollution and income status was narrowed since 1990 for the high PMH groups. Countries with a higher level of PMH had less significant differences in U5MR between low- and middle-income groups than those with a lower level of PMH. The development of PMH-related gradient in child health is not consistent thoroughly. Before 2000, boys experienced a sharper decline in PMH-related gradient in health than girls did. The decomposition shows that the changes in PMH-gradient in child health were mainly caused by changes in the return to risk factors. The mental health of female population matters more in child health outcomes.

**Conclusion:**

Although the U5MR converges across countries, the reason varies. The PMH gradient in child mortality is mainly explained by the change in the return to risk factors. The PMH-gradient health disparity in boys is larger than that in girls in 2019, which indicates that boys’ health may be more vulnerable to the development of PMH recently. The findings remind us that we need to pay attention to the hidden reasons for the growth of disparity. It also suggests that improving PMH has a great impact on reducing PMH-related health disparity, especially for boys. Our research contributes to the understanding of the transition of PMH-related health disparity in U5MR and provides policy implications for reducing gender disparity in child health.

**Supplementary Information:**

The online version contains supplementary material available at 10.1186/s12889-022-14530-w.

## Key messages

The difference in under five mortality rate (U5MR) between countries with different levels of air pollution and income status was narrowed since 1990 conditioning on population mental health (PMH) status.

The development of PMH-related gradient on child health is “inverted-V” shaped.

Boys experienced a sharper decline in PMH related gradient in health than girls did.

The changes in PMH-gradient in child health were mainly caused by changes in the return to risk factors.

PMH of the female population matters more in child health outcomes.

## Background

Achieving the goal of reducing child mortality remains a challenge. In 2019, the world average mortality rate for children under 5 years old (U5MR) per 1000 live births was 37.7 [1]. To achieve the target 3.2 of Sustainable Development Goals (SDGs) [2], an additional 12.7 thousandths need to be reduced. This rate of decrease (an annual reduction rate at 3.7% calculated as compound interest rate) is almost equal to the one that had been experienced in the previous 11 years before 2019. Globally, there are 71 (34.47%) regions or countries that have not reached the world’s average U5MR, among which low- and lower middle-income countries account for 61.42 and 43.2% of regions or countries that have not reached the U5MR target of SDGs 3.2.

To help improve U5MR, it is critical to analyze the drivers of child health. Existing literature has identified a variety of factors in explaining the difference in children’s health. The most highlighted factors are environment [3–6], education [7, 8], and income [9–14]. For example, of all the environmental factors, air pollution is regarded to be most strongly associated with infant mortality and U5MR [4]. In a study using child individual-level data and geographic air quality information from 1989 to 2000 in California, the air pollutants such as concentration of particulate matter (PM10) were found to be positively associated with child health through a policy shock [6]. Compared with other air pollutants such as CO and NO_2_, PM2.5 was associated with the highest infant mortality and U5MR, followed by PM10 [4]. Since education, especially parental education, is associated with better health behaviors and social resources, it is also regarded as one of the key factors affecting child health [8]. Studies that normally applied the difference-in-difference (DID) method and aimed at evaluating certain policy shocks reported that better parental education could reduce the incidence of low birth weight and fetal death [15]. In both developed countries, such as the United States [12], and developing countries, such as India (a country that contributes to the volume of deaths of children under five significantly [16]), income and education were found to be highly correlated with children’s health.

In addition to these most prominent factors, mental health should be taken into consideration. As guided by World Health Organization (WHO), there is “no health without mental health” [17]. Population mental health (hereinafter PMH) is not only a population health issue, it also influences “the progress towards the achievement of several Millennium Development Goals, such as … reduction of child mortality” [18]. In this regard, one of the important research topics is the association between maternal mental health and child outcomes [19–22], given that a mother’s mental health is one of the earliest impact factors in a child’s life [22]. In fact, several clinical studies showed that children of parents with depression had higher medical utilization and higher risk of mental health disorders than those of parents without depression [23]. However, in comparison with intensive studies of education, income, and environment on child health, discussion about the impact of PMH on child health is scarce. Existing discussions on PMH and child health have mainly focused on high-income countries, but a series of studies suggested that the relationship between mental health and child health might be different in low- and middle-income countries (LMICs) from that in high-income countries, considering the high prevalence of mental health disorders in LMICs [20]. These findings directly suggest the importance of examining inequalities in children’s health across the mental health gradient at the aggregate population level.

This study takes the PMH-gradient as the key research point and explores the relationship between education, environment, income, health behavior, and child health using national-level data. This approach distinguishes from the previous studies that discuss the education- [24–27] and income- [28–30] gradient in adult (or child) health at the micro level. By focusing on the PMH-related disparity in child health, this study distinguishes it from the conventional health inequality studies that used the concentration index (CI) as the key measurement [31–33]. Instead, this study discusses the changing PMH-gradient in children over time and explores sources of PMH-related health gradient in children.

To examine the PMH-gradient in child health, this study constructs a national-level dataset from 1990 to 2019, describes the PMH-related gradient in child health, and decomposes its impact using a quasi-Oaxaca-Blinder (OB) decomposition [34, 35] strategy. Thus, to observe the extent to which the change of the gaps in U5MR between high and low PMH status is due to the returns to investment in risk factors, or due to the change of the levels of these factors.

## Methods

### Data source

Data used in this study were obtained from the World Bank World Development Indicators (WDI) Database, the Penn World Table 10.0 (PWT100) [36], and Institute for Health Metrics and Evaluation (IHME) Database. Due to data availability, the raw data in use was an unbalanced panel starting from 1990 to 2019, and the analysis was performed with data using ten-year intervals. After we checked the completeness of the data and removed countries with missing data, 92 countries were included in the analysis. If countries had missing data, we used a linear extrapolation approach to impute the missing values. For example, if a country did not record the value of GDP per capita in 2000, we used the non-missing data points during the period of 1990–2019 in the country to fit a simple linear regression model to estimate the value for 2000.

### Measurement

#### Mortality rate under five (U5MR)

U5MR was measured in terms of the number of deaths among children under five per 1000 live births. The data were obtained from the WDI database. Given that the gender difference of U5MR is more sensitive than the infant mortality rate in capturing the effect of gender discrimination, we analyzed the U5MR for boys and girls separately. We collected the U5MR from 1990 to 2019 and constructed a panel data set with five-year intervals in order to be consistent with other indicators.

### Impact factors: PMH, socioeconomic status, environment, health behavior, urbanization, water and sanitation, healthcare level, HIV prevalence

PMH was measured by Deaths per 100,000 population due to population mental disorders. The data were obtained from IHME. Countries were classified into two PMH groups according to the percentile of Deaths per 100,000 population. Countries with Deaths per 100,000 population below the median were considered to have a low level of mental disorder burden (labeled as the low PMH group). Otherwise, the countries were considered to have a high level of mental disorder (labeled as the high PMH group). Therefore, the low PMH group referred to the group with a better mental health condition, while the high PMH group represented the group with worse mental health status.

We used two indicators as measurements of socioeconomic status for countries, including education and gross domestic product (GDP) per capita. Our measurement of education was derived from the human capital variable in the PWT100. The PWT database is a well-known database that studies the economic progress and capital stock (including human capital) in each country. The human capital variable is derived from average years of schooling. Therefore, average years of schooling can be derived from an inverse procedure (For more detail, please visit PWT100). GDP per capita in the 2015 USD was used as the measurement of the income status of a country. The data were obtained from WDI.

According to the literature, among numerous environmental factors, air pollution was highly correlated with U5MR [4]. Considering the data availability, air pollution was mainly referred to outdoor air pollution. The outdoor air pollution was measured by the mean annual exposure of fine particulate matter (PM2.5). Although the indoor population was also associated with the child mortality rate, we could not identify and obtain relevant data at the national level for the analysis. Thus, the air pollution in this paper was referred to outdoor air pollution, and PM 2.5 was the measurement of outdoor air pollution. A study showed that PM2.5 has the highest fatality rate among air pollutants that affect a child health, followed by PM10 [4]. The smaller particulate matter is, the easier it could reach to lung cell [6], and the damage is irreversible. The PM2.5 exposure was calculated by “weighting annual concentrations of PM2.5 by population in both urban and rural areas” according to the WDI database. Countries with PM2.5 below the quintile were considered to have a lower level of air pollution. Otherwise, the countries were considered to have a high level of air pollution. The data were obtained from WDI and it started from 1990 with five-year intervals. Countries were classified into either the high or low air pollution group.

We also collected data on the smoking rate and alcohol uptake as proxies of individuals’ health behaviors. The smoking rate was measured by the prevalence of current tobacco use among adults, and alcohol uptake was measured by the consumption of pure alcohol per capita over the age of 15. The same as previous measures, countries were divided into two groups based on quantiles. These two indicators were obtained from WDI. In addition, we controlled for a series of impact factors such as water and sanitation, urbanization, healthcare level, and HIV prevalence to exclude other influences on child mortality. Water and Sanitation were measured as the share of population accessing basic water and sanitation services. Urbanization was proxied by the share of urban population. Healthcare level was measured as the number of physicians per 1000 population. And HIV prevalence was measured as the share of the population (among the population aged 15–49) with HIV infection. All these indicators were from the WDI database.

### Ethical considerations

The data used in this study are publicly available, and ethical approval is not applicable.

### Statistical analysis

According to the determinants of health introduced by WHO [37], U5MR was summarized by the status of the PMH, socioeconomic status, environmental, health behavior, basic water and sanitation services, urbanization, healthcare level, and HIV prevalence. Additionally, we examined the extent to which those risk factors contributed to the changes in U5MR by using a quasi-OB decomposition [34, 35]. The analytical logic was to decompose the change in health disparity that was related to PMH status into two sources: the change in the level of health risk factors and the change in the return to risk factors.

The benchmark estimation equation is:

1 $$\ln \left[U5{MR}_i\left|\left(\textrm{sample}=\textrm{t}\right)\right.\right]={\beta}_0+{\beta}_1\ {PMHGRP}_i+H\ {Controls}_i^{\prime }+{\varepsilon}_i,$$

*U*5*MR*_*i*_|(sample = t) represents U5MR at year *t* for country *i*; *PMHGRP*_*i*_ equals to 1 if country *i* at year *t* is in high PMH group, and 0 in low PMH group. *Controls*_*i*_ is the control variable vector including education, GDP per capita, PM2.5, smoking status, alcohol behavior, basic water and sanitation services, urbanization, healthcare level, HIV prevalence rate, and regional fixed effect. H is the coefficient vector, and *ε*_*i*_ is the residual term.

We estimated Eq. (1) in year τ cross-sectionally. Then we used the estimated coefficients at year τ to predict the U5MR in year t by using the control variables at year t (Eq. (2)). Note that τ does not necessarily equal t. The estimated log form of U5MR at year t is:

2 $$\ln \left({\left.{\hat{U5 MR}}_i\right|}_{coef\ sample=\tau}^{data\ sample=t}\right)={\beta}_0^{\tau }+{\beta}_1^{\tau }{PMHGRP_i}^t+{Controls_i^t}^{\prime }\ {\textrm{H}}^{\tau }$$

Equation (2) allowed us to focus on the influence of mental health conditional on the control variables of each country. Then the estimated U5MR were estimated by exponentiating the results from Eq. (2), which is:

3 $${\left.{\hat{U5 MR}}_i\right|}_{coef\ sample=\tau}^{data\ sample=t}={e}^{\left({\beta}_0^{\tau }+{\beta}_1^{\tau }{PMHGRP_i}^t+{Controls}_i^t\prime {\textrm{H}}^{\tau}\right)}={e}^{\beta_1^{\tau }{PMHGRP_i}^t}\bullet \Theta$$

where $$\Theta ={e}^{\left({\beta}_0^{\tau }+{Controls}_i^t\prime {\textrm{H}}^{\tau}\right)}$$.

PMH (*PMHGRP*_*i*_) is a dummy variable which equals 1 if a country is in the high level PMH group, i.e. worse mental health group, thus the corresponding predicted U5MR was estimated as ${\left.{\hat{U5 MR}}_i\right|}_{coef\ sample=\tau}^{data\ sample=t}\left( PMHGRP=1\right)={e}^{\beta_1^{\tau }}\bullet \Theta$, and the average of multiple predicted U5MR was $\overline{\hat{U5 MR}}{\left|{}_{coef\ sample=\tau}^{data\ sample=t}\left( PMHGRP=1\right)=\frac{1}{N}{\sum}_i{\hat{U5 MR}}_i\right|}_{coef\ sample=\tau}^{data\ sample=t}\left( PMHGRP=1\right)$, where N is the number of country. Then, for example, the ${\left.\overline{\hat{U5 MR}}\right|}_{coef\ sample=2005}^{data\ sample=1990}\left( PMHGRP=1\right)$ and ${\left.\overline{\hat{U5 MR}}\right|}_{coef\ sample=2005}^{data\ sample=2005}\left( PMHGRP=1\right)$ were used to compare changes in U5MR due to risk factors from 1990 to 2005 in the high PMH group. To calculate the average predicted U5MR, we conducted a bootstrap procedure through 200 replications. In each replication, we randomly draw 70% sample for the prediction.

Taking the low PMH group as the reference group, the ratio of the mean of the predicted U5MR in the high PMH group to that in the low PMH group and (Eq. (4)) captures the PMH-related gradient in U5MR. A ratio above one means the average U5MR is higher in the high PMH group [24]. By using data at different time points, we estimated the value of ${\left.\hat{r}\right|}_{coef\ sample=\tau}^{data\ sample=t}$ (referred to $\hat{\textrm{r}}$ for simplicity) to examine the change in PMH-related U5MR gradient.

4 $${\left.\hat{r}\right|}_{coef\ sample=\tau}^{data\ sample=t}=\frac{\overline{{\left.\hat{U5 MR}\right|}_{\tau}^t}\left( PMHGRP=1\right)}{\overline{{\left.\hat{U5 MR}\right|}_{\tau}^t}\left( PMHGRP=0\right)}=\frac{{\left.\frac{1}{N_1}{\sum}_1^{N_1}{\hat{U5 MR}}_i\right|}_{\tau}^t\left( PMHGRP=1\right)}{{\left.\frac{1}{N_2}{\sum}_1^{N_2}{\hat{U5 MR}}_i\right|}_{\tau}^t\left( PMHGRP=0\right)}$$

All the above statistical analyses are done with the statistics software Stata 16.

## Results

### Descriptive statistical analysis

#### PMH and child health

**Fig. 1:**
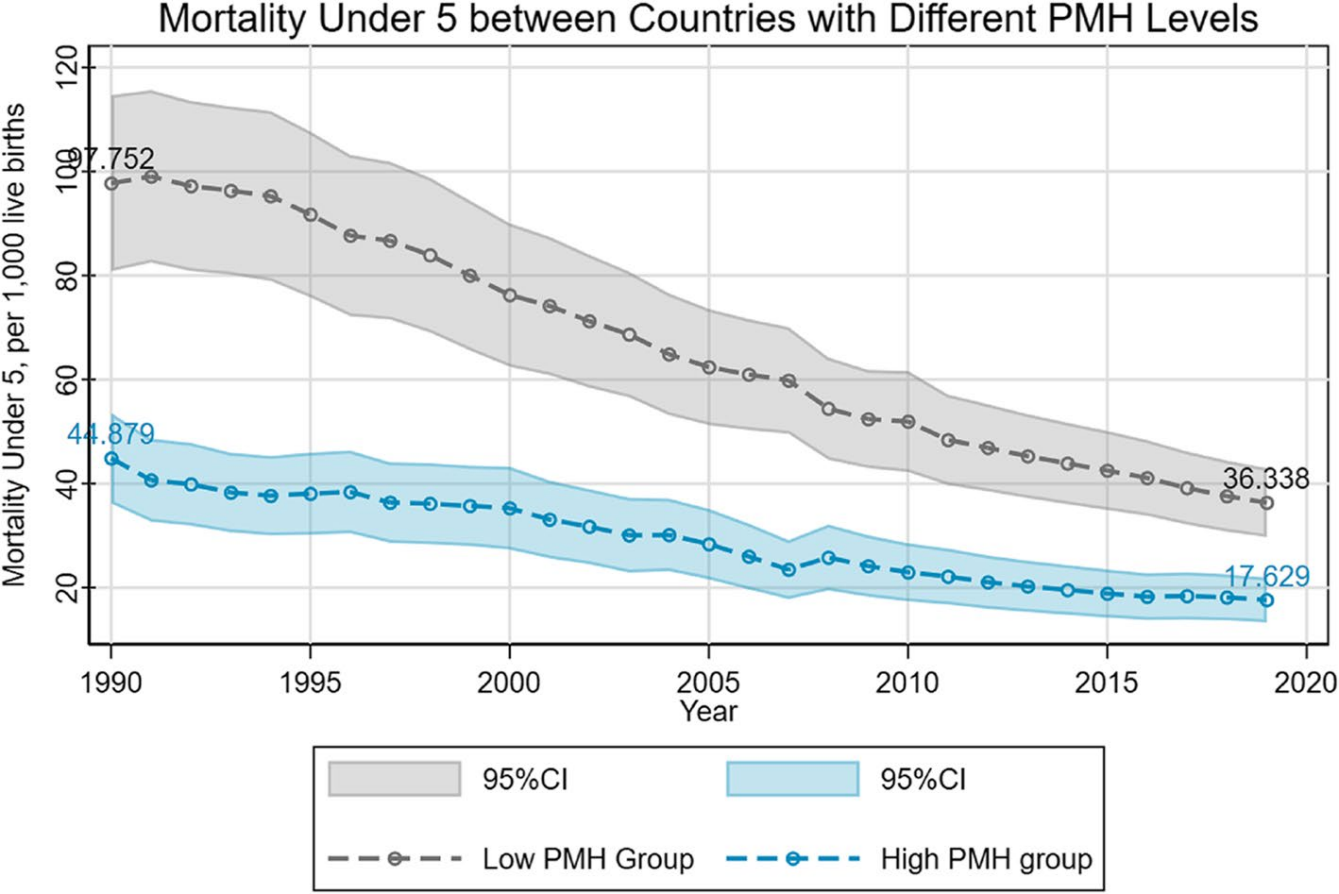
Mortality under 5 in mental health gradient

There was a significant difference in U5MR between countries with different PMH statuses. Countries with a low PMH status had a higher U5MR than countries with a high PMH status disorders. In our sample, the gap peaked in 1990, yielding a value of approximately 5.287 percentage points in U5MR. However, this gap gradually narrowed over time (Fig. 1). Generally, countries with a low level of mental disorder death rate were less wealthy in terms of GDP per capita, had a lower level of smoking and alcohol consumption, but had a higher level of PM2.5 (Fig. A1 in Appendix).

#### U5MR by environment group

Figure A2 and A3 in Appendix shows the relationship between U5MR and PM2.5. The level of PM2.5 was positively associated with U5MR, conditional on PMH groups or income groups. However, in low-income countries, the correlation between PM2.5 and U5MR was weaker, which suggests that there existed other factors that affect U5MR than PM2.5.

**Fig. 2:**
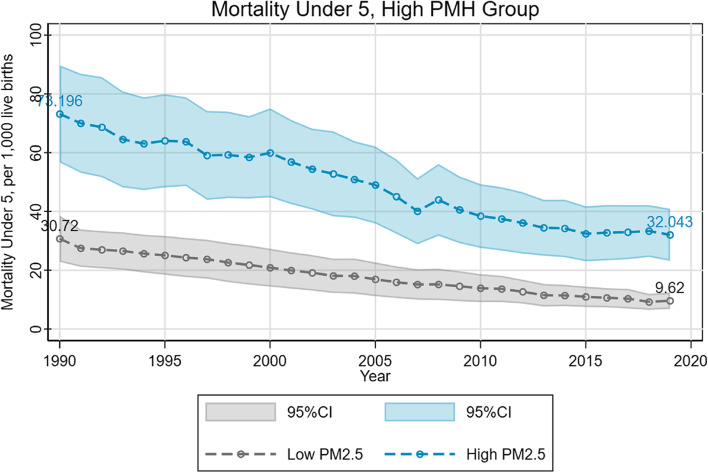
Environment-mortality gradient in high PMH group

**Fig. 3:**
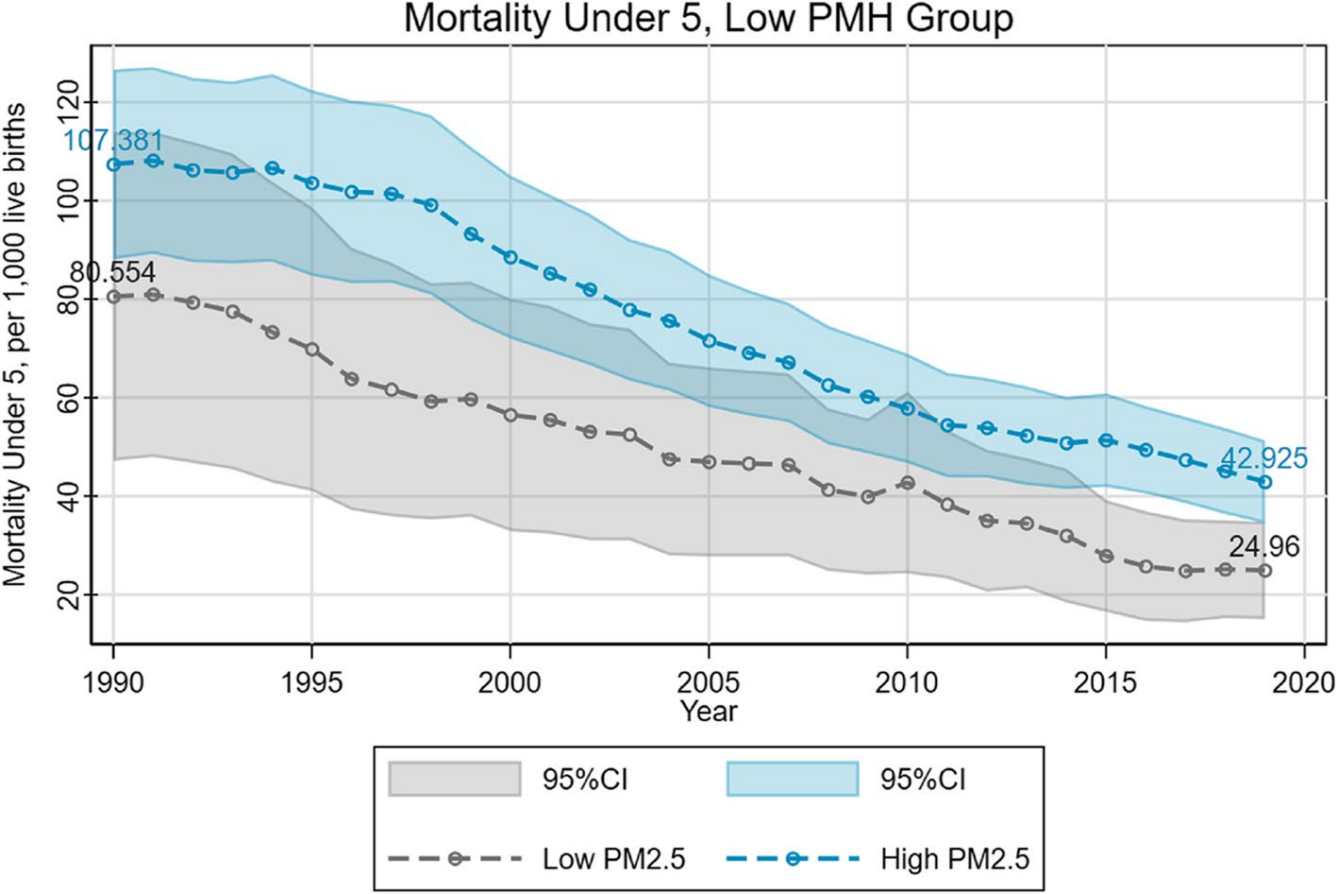
Environment-mortality gradient in low PMH group

We found that the difference in U5MR between the low and high PM2.5 groups varied conditionally on the PMH status. For countries with a high level of mental disorders, U5MR in higher PM2.5 exposed countries was significantly higher than that of less exposed countries (Fig. 2). The gap observed in 1990 reached 42.48 per 1000 live births, which means that the number of deaths per 1000 children before their fifth birthday in high-pollution countries increased by 42 compared to low-pollution countries. This gap was narrowed to 22.42 per 1000 live births in 2019. However, in the countries with a low level of mental disorders, the difference in U5MR between the countries with different levels of PM2.5 exposure is much smaller (Fig. 3).

#### U5MR by education and income group

Table 1 shows that in the high PMH group, the mortality rate of low-income countries in 1990 was 6.9 percentage points higher than high-income countries. The absolute gap decreased to 2.4 percentage points in 2019. Relatively, the mortality ratio between low-and high-income countries rises from 8.09 (161.57/19.97) in 1990 to 8.38 (63.54/7.58) in 2019.

**Table 1:** U5MR by income and PMH level (per 1000 live births)

Low PMH Group						
Year	Low Income		Middle Income		High Income	
1990	161.57	(142.17, 180.97)	55.83	(37.49, 74.18)	19.97	(15.25, 24.69)
2000	136.50	(122.77, 150.22)	39.34	(25.54, 53.15)	12.51	(9.56, 15.45)
2010	91.57	(79.80, 103.34)	25.09	(17.58, 32.60)	9.25	(6.60, 11.91)
2019	63.54	(55.04, 72.03)	17.22	(15.82, 20.01)	7.58	(5.45, 9.71)
High PMH Group						
Year	Low Income		Middle Income		High Income	
1990	92.60	(71.35, 113.85)	48.75	(39.75, 57.75)	15.57	(10.57, 20.57)
2000	70.14	(52.72, 87.57)	43.96	(30.79, 57.14)	10.83	(6.47, 15.19)
2010	51.32	(38.45, 64.18)	24.02	(16.95, 31.09)	8.74	(3.19, 14.29)
2019	39.56	(29.03, 50.08)	20.21	(13.55, 26.87)	4.94	(3.94, 5.93)

The income category is based on the percentile of GDP per capita (2015 USD). The top 33.33% is regarded as the high-income group, the middle 33.33% as the middle-income group, and the bottom 33.33% as the low-income group

During 1990 and 2019, the mortality rate in the low PMH group decreased about 9.84 percentage points in the low-income group and decreased by 3.86 percentage points in the middle-income group. The absolute gap in U5MR between low- and middle-income countries reduced from 10.57 percentage points to 4.63 percentage points, which was greater than that in the high PMH group. The PMH-mortality gradient by gender is shown in Figs. A4 and A5 in Appendix. For the low PMH group, the mortality rate in the low-income group in 2019 was 63.54‰, which was 60.62% more than that in the high PMH group. This disparity was less salient when we compared U5MR in countries with higher income between the two PMH groups.

### PHM-gradient and U5MR

Table 2 shows the results from the OLS estimation of Eq. (1). Overall, U5MR has a positive relationship with PMH burden, air pollution, smoking and alcohol consumption, urbanization, and HIV prevalence, but had an inverse relationship with socioeconomic status and basic sanitation services. In general, the effect of PMHGRP was significant, and the U5MR is 20.2% higher in countries with high PMH status in 1990, this difference decreased to 13.3% in 2019. We also used the DALYs of mental disorder to represent the burden of PMH. The results were in Table A8 in Appendix. Figure A6 in Appendix shows the general additive model (GAM) analysis of U5MR against DALYs due to mental disorders. It showed that in developed countries, as DALYs from mental disorders increased, the U5MR increased. However, the relationship between mental disorder and U5MR in LMICs was mixed.

**Table 2:** The regression results on determinants of U5MR in each time point

Variables	(1)	(2)	(3)	(4)	(5)
	1990	2000	2005	2010	2019
	Ln(U5MR)	Ln(U5MR)	Ln(U5MR)	Ln(U5MR)	Ln(U5MR)
PMHGRP(=1)	0.202**	0.226**	0.195*	0.130	0.133
	(2.197)	(2.514)	(1.749)	(1.094)	(1.102)
Ln(Educ)	−0.942***	− 0.944***	− 0.777***	− 0.878**	− 1.040***
	(−3.083)	(−3.554)	(−2.765)	(−2.608)	(− 3.169)
Ln(GDP per capita)	− 0.477***	− 0.459***	− 0.459***	− 0.445***	− 0.353***
	(−7.459)	(−8.780)	(− 7.473)	(−6.316)	(−5.191)
Ln(PM2.5)	0.241**	0.167*	0.154	0.153	0.162
	(2.287)	(1.677)	(1.378)	(1.282)	(1.602)
SmokGRP	0.0426	0.0934	0.157	0.108	−0.0266
	(0.439)	(1.117)	(1.539)	(1.158)	(−0.230)
AlcohGRP	0.105	0.104	0.0895	0.0436	0.00838
	(1.016)	(1.153)	(0.868)	(0.291)	(0.0710)
Ln(Basic Water)	−0.225	0.0956	0.205	0.163	0.302
	(−1.035)	(0.442)	(0.745)	(0.386)	(0.506)
Ln(Basic Sanitation)	−0.199**	−0.104	− 0.157	−0.210	− 0.120
	(−2.373)	(− 1.526)	(− 1.524)	(− 1.325)	(− 0.661)
Ln(Urban Pop.)	0.231	0.0741	0.0877	0.183	0.185
	(1.563)	(0.498)	(0.531)	(1.019)	(0.871)
Healthcare Level	0.0272	−0.0166	−0.0254	−0.0530	− 0.102**
	(0.430)	(−0.214)	(−0.312)	(− 0.717)	(−2.625)
Ln(HIV)	0.0410	0.0744*	0.0848*	0.109*	0.122**
	(1.024)	(1.893)	(1.823)	(1.893)	(2.374)
Region	Y	Y	Y	Y	Y
Observations	80	92	92	92	90
Adj. R-squared	0.909	0.920	0.901	0.887	0.881
F	NA	NA	NA	NA	NA

Robust t-statistics in parentheses. Column (1) to Column (5) are the regression results from year 1990, 2000, 2005, 2010, 2019, respectively

*** *p* < 0.01, ** *p* < 0.05, * *p* < 0.1

As to the control variables, we found that, for example, a 1% increase in PM2.5 was associated with a 0.241% increase in U5MR and a 1% increase in GDP per capita was associated with a 0.477% reduction in U5MR in 1990. The regression results disaggregated by gender are shown in Table A1 and A2 in Appendix, and Table A5 shows the countries included in the regression analysis. Including the mental health variable in the regression, Table A3 and A4 provides us with a clear picture that mental health condition affected U5MR more in the female population (which is consistent with the findings from previous studies).

A T-test showed the estimated U5MR differential between countries with and without a low PMH environment is significant at 1% level. We present the $\hat{\textrm{r}}$ (the PMH-related U5MR gradient, which is significant at 0.001 level) calculated from the estimated mortality rate using Eq. (4) in Tables 3, 4, 5. Holding the row constant, moving across each column, we observe the changes of $\hat{\textrm{r}}$ due to the change of the level of risk factors including education, income, environment, health behavior, basic water, basic sanitation, urbanization, healthcare level, and HIV prevalence when the coefficients of the risk factors remained unchanged. The values in the same column show the change of $\hat{\textrm{r}}$ due to the change in the coefficient of risk factors (return on the investment in addressing the risk factors) while keeping the level of risk factors at the same level across different times. Tables A6 and A7 provide the calculated $\hat{\textrm{r}}$ using PMH by gender.

**Table 3:** The ratio of average U5MR between countries in high PMH group and those with low PMH group

		Data	Data	Data	Data	Change in data
		1990	2000	2010	2019	*Δ*_1990 − 2019_
Correlation 1990		**1.0837**	1.0540	1.0627	1.0667	−0.0171
		**(0.0013)**	(0.0009)	(0.0010)	(0.0012)	(0.0018)
Correlation 2000		1.1003	**1.0680**	1.0764	1.0804	−0.0199
		(0.0015)	**(0.0010)**	(0.0012)	(0.0014)	(0.0020)
Correlation 2010		1.0552	1.0368	**1.0421**	1.0444	−0.0108
		(0.0009)	(0.0006)	**(0.0007)**	(0.0008)	(0.0012)
Correlation 2019		1.0961	1.1007	1.0964	**1.0470**	−0.0138
		(0.0006)	(0.0005)	(0.0005)	**(0.0008)**	(0.0012)
Change in correlation	*Δ*_1990 − 2019_	−0.0230	−0.0130	− 0.0176	−0.0197	**− 0.0368**
		(0.0005)	(0.0003)	(0.0004)	(0.0004)	**(0.0016)**

The under-five mortality ratio is calculated as $\hat{\textrm{r}}={\overline{\hat{U5 MR}}}_{PMHGRP=1}/{\overline{\hat{U5 MR}}}_{PMHGRP=0}$, where $\overline{\hat{U5 MR}}$ stands for the mean of estimated U5MR. And the baseline prediction of $\hat{U5 MR}$ uses PMH status, education, environment, GDP per capita, health behavior, water and sanitation, urbanization, healthcare level, and HIV prevalence. The reported ratios in Table 3 are computed as the mean of $\hat{\textrm{r}}$ across 200 bootstrap replications, standard errors in parentheses. In each bootstrap replication, we randomly draw 70% of the total sample

**Table 4:** The ratio of average U5MR (female) between countries in high PMH group and those with low PMH group

		Data	Data	Data	Data	Change in data
		1990	2000	2010	2019	*Δ*_1990 − 2019_
Correlation 1990		**1.0823**	1.0527	1.0615	1.0655	−0.0169
		**(0.0013)**	(0.0009)	(0.0010)	(0.0012)	(0.0018)
Correlation 2000		1.1012	**1.0681**	1.0768	1.0810	−0.0202
		(0.0015)	**(0.0011)**	(0.0012)	(0.0014)	(0.0021)
Correlation 2010		1.0514	1.0342	**1.0392**	1.0413	−0.0102
		(0.0008)	(0.0005)	**(0.0006)**	(0.0007)	(0.0011)
Correlation 2019		1.0593	1.0400	1.0440	**1.0458**	−0.0135
		(0.0008)	(0.0006)	(0.0007)	**(0.0008)**	(0.0011)
Change in correlation	*Δ*_1990 − 2019_	−0.0230	−0.0127	− 0.0175	−0.0197	**− 0.0366**
		(0.0006)	(0.0003)	(0.0004)	(0.0004)	**(0.0016)**

The under-five mortality ratio is calculated as $\hat{\textrm{r}}={\overline{\hat{U5 MR}}}_{PMHGRP=1}/{\overline{\hat{U5 MR}}}_{PMHGRP=0}$, where $\overline{\hat{U5 MR}}$ stands for the mean of estimated U5MR. And the baseline prediction of $\hat{U5 MR}$ uses PMH status, education, environment, GDP per capita, health behavior, water and sanitation, urbanization, healthcare level, and HIV prevalence. The reported ratios in Table 4 are computed as the mean of $\hat{\textrm{r}}$ across 200 bootstrap replications, standard errors in parentheses. In each bootstrap replication, we randomly draw 70% of the total sample

**Table 5:** The ratio of average U5MR (male) between countries in high PMH group and those with low PMH group

		Data	Data	Data	Data	Change in data
		1990	2000	2010	2019	*Δ*_1990 − 2019_
Correlation 1990		**1.0859**	1.0558	1.0645	1.0685	−0.0174
		**(0.0013)**	(0.0009)	(0.0010)	(0.0012)	(0.0018)
Correlation 2000		1.0990	**1.0674**	1.0755	1.0794	−0.0195
		(0.0014)	**(0.0010)**	(0.0011)	(0.0013)	(0.0020)
Correlation 2010		1.0564	1.0377	**1.0431**	1.0454	−0.0110
		(0.0009)	(0.0006)	**(0.0007)**	(0.0008)	(0.0012)
Correlation 2019		1.0639	1.0432	1.0476	**1.0494**	−0.0144
		(0.0009)	(0.0007)	(0.0007)	**(0.0008)**	(0.0012)
Change in correlation	*Δ*_1990 − 2019_	−0.0220	−0.0126	− 0.0169	−0.0191	**− 0.0220**
		(0.0005)	(0.0003)	(0.0003)	(0.0004)	**(0.0005)**

The under-five mortality ratio is calculated as $\hat{\textrm{r}}={\overline{\hat{U5 MR}}}_{PMHGRP=1}/{\overline{\hat{U5 MR}}}_{PMHGRP=0}$, where $\overline{\hat{U5 MR}}$ stands for the mean of estimated U5MR. And the baseline prediction of $\hat{U5 MR}$ uses PMH status, education, environment, GDP per capita, health behavior, water and sanitation, urbanization, healthcare level, and HIV prevalence. The reported ratios in Table 5 are computed as the mean of $\hat{\textrm{r}}$ across 200 bootstrap replications, standard errors in parentheses. In each bootstrap replication, we randomly draw 70% of the total sample

The bold figures on the main diagonal show the ratios estimated by using the concurrent changes of risk factors and coefficients. For example, in 1990, U5MR in the high PMH countries was 8.37% (1.0837–1) more than that in the low PMH ones (Table 3). In 2019, the female U5MR of the high PMH countries was 4.58% higher than that of the low PMH peers (Table 4). This number was 4.94% for males (Table 5). The overall decrease in the PMH-related U5MR gradient from 1990 to 2019 was 3.68 percentage points, with 3.66 and 3.65 percentage points for females and males, respectively.

The trend in the PMH-related gradient in U5MR was not consistent. There was a decrease in the ratio of the U5MR between the two PMH groups in the first 20 years since 1990, but the PMH-gradient in U5MR increased in the next decade. Such a trend of PMH-related gradient in U5MR occurred in both genders (Tables 4 and 5). Overall, the PMH-related U5MR gradient declined slightly more for girls (3.66 percentage points) than for boys (3.65 percentage points) from 1990 to 2019. We can also find that the PMH-related U5MR gradient increased by 0.66 percentage points in the 1990s for girls and by 0.63 percentage points for boys. This means that for the last decade of the 1990–2019 period, girls’ PMH-related U5MR gradient climbed more rapidly than boys. But for the first two decades, the gap of U5MR declined faster in girls (4.32 percentage points) than in boys (4.27 percentage points).

Row (Correlation 1990) in Table 3 shows changes in $\hat{\textrm{r}}$ due to the change of risk factors while holding the impact coefficients constant at that in 1990. For instance, the PMH gradient in U5MR decreased by about 1.71 percentage points when the estimated coefficient stayed at the 1990 level while the risk factors were at the 2019 level. The 1.71 percentage point decrease resulted in 6.67% higher U5MR in the high-PMH group compared with that in the low-PMH counterparts, which exceeded the actual ratio between the low- and high- PMH groups (1.047).

When evaluating value within a column, which represents different returns in addressing risk factors changes over time by assuming the same level of risk factors at the 1990 level (Column (Data 1990) Table 3), the PMH-related gradient in U5MR reduced about 2.3 percentage points from 1990 to 2019. The decrease was smaller than the actual decline of 3.68 percentage points (accounting about 62.49% in the reduction). Such a result shows that the decrease in PMH-related U5MR gradient was mainly due to the change in the impact coefficients of risk factors. It was consistent in both genders.

The $\hat{\textrm{r}}$ of mortality rate in boys (1.0859) was slightly higher than that in girls (1.0823) in year 1990, however, the disparity in PMH-related gradient in boys decreased to 1.0494 in 2019, which is 0.36 percentage points larger than that in girls (1.0458). In another word, the disparity in PMH-mortality gradient was slightly more severe in boys than girls in 2019. The pattern was consistent when using the PMH of the female population as the key variable in child health disparity analysis (Table A6 in Appendix). What’s more, in the 2010s, the estimated child health gradient associated with PMH was greater when female mental disorders were considered as the key explanatory variable, as opposed to using male mental health status as a key explanatory variable.

## Discussion

This study presents a retrospective country-level analysis of PMH-related gradient in U5MR for 30 years and shows that the PMH-gradient in U5MR changes over time. The environment-related disparity in U5MR is more significant in the high PMH group. The gap in child mortality between low- and middle-income groups converges faster in the low PMH group.

Following a decomposition approach that is closely related to OB decomposition [24], we focus on the PMH-related gradients in U5MR. This approach decomposes the changes in PMH-related gradient in child health into two sources: the change of the return to risk factors (the changes in coefficients), and the change in the level of risk factors. The PMH (in female population)-related gradient in children’s health shows an “inverted-V” shape in both girls and boys.

The study shows that trends in the risk factors mainly accounted for the change in gradient associated with PHM in U5MR during the sample period. From 1990 to 2019, changes in levels of risk factors (such as an increase in the urbanization rate) would lead to a smaller change in PMH-related disparity in U5MR if the impact of each risk factor remains unchanged. In other words, changes in levels in risk factors over the past 30 years did not contribute to a salient decrease in PHM-related childhood health disparity. The same pattern applies to both boys and girls.

We also found that the gap in U5MR between countries with and without a high PMH environment declined by 3.68 percentage points, whereas the corresponding change in the return to risk factors predicted a 2.3-point incline. This means the rise in PMH-related gradient in U5MR is mainly due to the changes in the return to risk factors, such as the differential return to education over time. Our findings show that instead of the changes in risk factors that raise the PMH-related gradient in U5MR, it is the return to PMH (conditional on risk factors) and changes in returns to education, income, healthcare level, health behaviors and water and sanitation services that play a major role in reducing PMH-related gradient in U5MR. For example, the impact of education and income, as well as healthcare, have increased over time for children.

While there has been an overall downward trend in the child health gradient associated with PMH over the past 30 years, the health disparity does not decrease in a consistent pattern over time. In the first 20 years of the study period, health disparity decreased, and then it began to climb up. From 2010 to 2019, the PMH-related gradient in child mortality increased by about 0.48 percentage points, and this change is mainly explained by the change in the return to risk factors. The main driver of the upward and downward change in PMH-related gradient in child health is primarily the evolution in the strength of the impact of risk factors. One of the possible explanations is that if risk factors were at a relatively high level, a small amount of investment might only cause subtle changes in levels. The magnitude of the impact of each impact factor on PMH-related gradient in child health has changed considerably and has become more influential. Taking education as an example, at the macro level, although the PMH status of a country is related to its development status (as discussed earlier) and countries at different stages of development have their own distribution of risk factors, the marginal role of education has increased substantially in the last 30 years due to knowledge spillover from globalized information exchange and purposeful promotion of health education by international organizations. At the same time, although environmental pollution and disease epidemics have a serious inverse impact on children’s health, the extent to which this impact may also be weakened due to the wider coverage of medical care and the advancement of medical technology. Therefore, changes in the influence of risk factors are more likely to explain the trends in PMH-related gradient in U5MR than changes in the volume of the factors themselves.

Over the past 30-year period, PMH-related health disparity in boys experienced a relatively larger fluctuation in than girls (around 2000), but after 2000, health disparity declined more for girls than for boys and came to a slightly lower level of disparity in 2019 than that in girls. The cause of this phenomenon needs to be further explored.

Studies showed that mental health issues, such as postpartum depression and anxiety, are closely linked to infant development [38, 39]. At the same time, studies have also shown that the gender of the baby has a direct impact on postpartum depression. There is evidence that having a girl baby is more associated with postpartum depression than a boy baby [40, 41], which may be due to, for example, a lack of family support (financial, physical and mental) [42]. And some evidences point out that compared with the baby girl, male infants are associated with increased incidence of postnatal depression [43], due to higher possibility of getting disease such as inflammation or pre-eclampsia [44]. In any case, families who suffer more mental health problems in early childhood and may cause a higher rate of adverse outcomes in child development. For example, a large body of literature reports that child malnutrition is associated with maternal depression in developing countries [45]. Studies show that the intellectual development of boys is affected by maternal depression in the first year postpartum [46], and infant boys with anxious mothers significantly reduced their motivation to interact with their mothers compared to girls with anxious mothers [47]. The developmental differences between boys and girls caused by this mental problem can be reflected in academic test scores at the age of 16 [48]. It is also important to note that boys are typically more active than girls, which leads to the possibility that boys are at a higher a risk than girls from certain conditions such as injuries. Considering that a child’s safety often depends on the care of his or her parents, if the parents are in good mental health, the risky behavior of the child can be effectively avoided. However, if the parents are suffered from mental disorders, they may be more easily distracted from the care, which would increase the likelihood of the child being exposed to risks.

In addition, our data shows that the absolute value of the correlation coefficient of depressive disorder DALYs (female) with U5MR (female) and U5MR (male) is 0.3377 and 0.3354, respectively. While the relevant value of depressive disorder DALYs in males is 0.4116 and 0.4124, respectively. By using anxiety disorder, we get a similar result, i.e. the absolute value of correlation coefficient of anxiety disorder DALYs (female) with U5MR (female) and U5MR (male) is 0.4228 and 0.4333, respectively. While the relevant value of anxiety disorder DALYs in males is 0.1504 and 0.1606, respectively.

Given the strong correlation between mental health disorders and infant outcomes, early intervention policies such as parental education, family visits, and enhanced community volunteerism in housekeeping and childcare should be considered.

Our finding, on the one hand, reminds us that we need to pay attention to the hidden reasons for the growth of disparity. On the other hand, it also shows that improving PMH status is essential in reducing PMH-related health disparity.

In sum, this paper contributes to the existing literature in two ways: firstly, it shows the macro landscape of PMH-related gradient in child health and provides evidence on the evolution of children’s health with socioeconomic status, environmental, health behavior, basic water and sanitation services, urbanization, healthcare level, and HIV prevalence conditional on PMH status. Secondly, this study uses the observed and predicted U5MR to examine the time-varying relationship between health and PMH and distinguishes the possible cause of the changes (changes in level of risk factors vs. changes in coefficients of risk factors).

One limitation is that we used quantiles when grouping samples according to PMH and control variables. It is less accurate since the impact of PMH and control variables such as environmental exposure on health may not follow a dose-response pattern. For example, above a certain threshold, environmental pollution represented by PM2.5 would have a significant impact on health. The quantile grouping does not distill such a threshold effect. In this study, we did not find any literature specifying a threshold for grouping. Our regression models show the consistent and expected signs for these two variables, suggesting a good validity of the models. This may mitigate the concerns of inaccurate grouping based on PMH and PM2.5. Our study presents disparity in U5MR associated with mental health, changes in such disparity over time, and the drivers of the changes. Another limitation of this study comes from the inability to represent in more detail how population mental health has differential effects on child health. This is largely due to the unavailability of relevant data. For example, we could not distinguish among various mental health disorders, such as prenatal and postnatal depressive conditions, anxiety, and suicide, which limits our further discussion of the drivers of the inequalities identified in the paper and possible interventions to mitigate inequalities. we had to rely on existing studies in the discussion to explain the results and possible mechanisms.

## Conclusion

This study decomposes the time-varying PMH-health gradient in children. In high PMH groups, the difference in child health among different environmental statuses converge from 1990 to 2019. The PMH-related gradient in child health changes over time. Considering socioeconomic status, environmental pollution, health behavior, basic water and sanitation services, urbanization, healthcare level, and HIV prevalence, child health disparity decreased from 1990 to 2010, but increased in 2010s. The cause of changes in PMH child health gradient has been consistent since 1990. The change in PMH-related under-five mortality disparity is explained primarily by the return on health risk factors. There is a gender difference in PMH-gradient, which suggests that the health of boys under five is more influenced by PMH. PMH-related health disparity declined more in girls than in boys after 2000. And the PMH-related health disparity in boys is slightly higher than that in girls, which also highlights that promoting population mental health status is important to reducing PMH-related child health disparity in boys. Lastly, PMH of female population matters more in child health.

## Supplementary Information

**Additional file 1..**

## Data Availability

The datasets used during the current study are available from the corresponding author on reasonable request.

## Abbreviations

U5MR: Mortality rate under five
PMH: Population mental health
SDGs: The target 3.2 of Sustainable Development Goals
WHO: World Health Organization
LMICs: Low- and middle-income countries
OB: Oaxaca-Blinder
WDI: World Bank World Development Indicators
PWT100: The Penn World Table 10.0
IHME: Institute for Health Metrics and Evaluation
GDP: Gross domestic product
PM2.5: Fine particulate matter
