# Supplementary material for: Population mental health matters child health disparity: a national level analysis
Source: BMC Public Health. 2022 Dec 17;22:2372. doi: 10.1186/s12889-022-14530-w (PMC9759864; doi:10.1186/s12889-022-14530-w)
Supplement: Supplementary file 1 — Additional file 1.. [file 12889_2022_14530_MOESM1_ESM.docx]

**Table A1 The regression results on determinants of U5MR (female) in each time point**

|  | (1) | (2) | (3) | (4) | (5) |
| --- | --- | --- | --- | --- | --- |
|  | 1990 | 2000 | 2005 | 2010 | 2019 |
| VARIABLES | Ln(U5MR) | Ln(U5MR) | Ln(U5MR) | Ln(U5MR) | Ln(U5MR) |
| PMHGRP(=1) | 0.202** | 0.231** | 0.195* | 0.122 | 0.130 |
|  | (2.170) | (2.564) | (1.749) | (1.030) | (1.088) |
| Ln(Educ) | -0.994*** | -1.017*** | -0.841*** | -0.933*** | -1.066*** |
|  | (-3.272) | (-3.848) | (-3.004) | (-2.741) | (-3.271) |
| Ln(GDP per capita) | -0.480*** | -0.456*** | -0.453*** | -0.440*** | -0.349*** |
|  | (-7.434) | (-8.775) | (-7.449) | (-6.278) | (-5.118) |
| Ln(PM2.5) | 0.255** | 0.174* | 0.161 | 0.157 | 0.166 |
|  | (2.413) | (1.764) | (1.447) | (1.317) | (1.640) |
| SmokGRP | 0.0462 | 0.0891 | 0.157 | 0.104 | -0.0204 |
|  | (0.467) | (1.049) | (1.525) | (1.113) | (-0.180) |
| AlcohGRP | 0.106 | 0.109 | 0.0971 | 0.0498 | 0.0101 |
|  | (1.013) | (1.196) | (0.941) | (0.337) | (0.0863) |
| Ln(Basic Water) | -0.224 | 0.100 | 0.210 | 0.164 | 0.262 |
|  | (-1.014) | (0.459) | (0.762) | (0.391) | (0.440) |
| Ln(Basic Sanitation) | -0.205** | -0.109 | -0.160 | -0.213 | -0.114 |
|  | (-2.424) | (-1.575) | (-1.532) | (-1.335) | (-0.617) |
| Ln(Urban Pop.) | 0.238 | 0.0792 | 0.0956 | 0.196 | 0.203 |
|  | (1.594) | (0.534) | (0.581) | (1.101) | (0.964) |
| Healthcare Level | 0.0248 | -0.0166 | -0.0262 | -0.0563 | -0.103*** |
|  | (0.398) | (-0.219) | (-0.326) | (-0.764) | (-2.665) |
| Ln(HIV) | 0.0443 | 0.0760* | 0.0867* | 0.110* | 0.120** |
|  | (1.081) | (1.895) | (1.845) | (1.869) | (2.345) |
| Region | Y | Y | Y | Y | Y |
| Observations | 80 | 92 | 92 | 92 | 90 |
| Adj. R-squared | 0.912 | 0.923 | 0.905 | 0.889 | 0.883 |
| F | . | . | . | . | . |

Note: *** p<0.01, ** p<0.05, * p<0.1. Robust t-statistics in parentheses. Column (1) to Column (5) are the regression results from year 1990, 2000, 2005, 2010, 2020, respectively.

**Table A2 The regression results on determinants of U5MR (male) in each time point**

|  | (1) | (2) | (3) | (4) | (5) |
| --- | --- | --- | --- | --- | --- |
|  | 1990 | 2000 | 2005 | 2010 | 2019 |
| VARIABLES | Ln(U5MR) | Ln(U5MR) | Ln(U5MR) | Ln(U5MR) | Ln(U5MR) |
| PMHGRP(=1) | 0.204** | 0.221** | 0.192* | 0.132 | 0.140 |
|  | (2.231) | (2.461) | (1.733) | (1.112) | (1.142) |
| Ln(Educ) | -0.902*** | -0.885*** | -0.727** | -0.832** | -1.023*** |
|  | (-2.939) | (-3.321) | (-2.577) | (-2.498) | (-3.105) |
| Ln(GDP per capita) | -0.475*** | -0.462*** | -0.462*** | -0.450*** | -0.356*** |
|  | (-7.457) | (-8.775) | (-7.493) | (-6.410) | (-5.189) |
| Ln(PM2.5) | 0.229** | 0.160 | 0.150 | 0.150 | 0.161 |
|  | (2.171) | (1.601) | (1.338) | (1.259) | (1.580) |
| SmokGRP | 0.0403 | 0.0968 | 0.157 | 0.111 | -0.0311 |
|  | (0.421) | (1.170) | (1.557) | (1.188) | (-0.264) |
| AlcohGRP | 0.105 | 0.100 | 0.0839 | 0.0396 | 0.00614 |
|  | (1.018) | (1.107) | (0.817) | (0.261) | (0.0516) |
| Ln(Basic Water) | -0.227 | 0.0918 | 0.199 | 0.165 | 0.335 |
|  | (-1.055) | (0.426) | (0.720) | (0.387) | (0.563) |
| Ln(Basic Sanitation) | -0.194** | -0.100 | -0.153 | -0.207 | -0.127 |
|  | (-2.320) | (-1.481) | (-1.511) | (-1.312) | (-0.705) |
| Ln(Urban Pop.) | 0.226 | 0.0709 | 0.0853 | 0.175 | 0.168 |
|  | (1.534) | (0.475) | (0.517) | (0.975) | (0.787) |
| Healthcare Level | 0.0290 | -0.0161 | -0.0243 | -0.0522 | -0.102** |
|  | (0.452) | (-0.205) | (-0.295) | (-0.704) | (-2.582) |
| Ln(HIV) | 0.0388 | 0.0736* | 0.0840* | 0.109* | 0.124** |
|  | (0.986) | (1.902) | (1.824) | (1.913) | (2.398) |
| Region | Y | Y | Y | Y | Y |
| Observations | 80 | 92 | 92 | 92 | 90 |
| Adj. R-squared | 0.907 | 0.917 | 0.899 | 0.886 | 0.879 |
| F | . | . | . | . | . |

Note: *** p<0.01, ** p<0.05, * p<0.1. Robust t-statistics in parentheses. Column (1) to Column (5) are the regression results from year 1990, 2000, 2005, 2010, 2020, respectively.

**Table A3 The regression results on determinants of U5MR in each time point, PMH in females**

|  | (1) | (2) | (3) | (4) | (5) |
| --- | --- | --- | --- | --- | --- |
| VARIABLES | 1990 | 2000 | 2005 | 2010 | 2019 |
| Panel A: Dependent Variable: U5MR | | | | | |
| PMHGRP(=1) | 0.107 | 0.240*** | 0.194* | 0.153 | 0.0732 |
|  | (1.084) | (2.736) | (1.701) | (1.328) | (0.559) |
| Ln(Educ) | -0.892** | -0.976*** | -0.778*** | -0.912** | -0.964*** |
|  | (-2.632) | (-3.586) | (-2.710) | (-2.635) | (-2.868) |
| Ln(GDP per capita) | -0.479*** | -0.473*** | -0.462*** | -0.446*** | -0.357*** |
|  | (-7.540) | (-9.130) | (-7.444) | (-6.319) | (-5.184) |
| Ln(PM2.5) | 0.251** | 0.177* | 0.154 | 0.157 | 0.167 |
|  | (2.453) | (1.808) | (1.367) | (1.329) | (1.647) |
| SmokGRP | 0.0560 | 0.0746 | 0.156 | 0.103 | -0.0364 |
|  | (0.561) | (0.873) | (1.529) | (1.114) | (-0.309) |
| AlcohGRP | 0.0950 | 0.130 | 0.0960 | 0.0500 | 0.0143 |
|  | (0.940) | (1.503) | (0.934) | (0.340) | (0.119) |
| Ln(Basic Water) | -0.232 | 0.0840 | 0.219 | 0.173 | 0.228 |
|  | (-1.023) | (0.403) | (0.804) | (0.409) | (0.378) |
| Ln(Basic Sanitation) | -0.186** | -0.0820 | -0.162 | -0.206 | -0.106 |
|  | (-2.231) | (-1.223) | (-1.574) | (-1.293) | (-0.580) |
| Ln(Urban Pop.) | 0.268* | 0.139 | 0.106 | 0.203 | 0.220 |
|  | (1.870) | (0.978) | (0.631) | (1.144) | (1.042) |
| Healthcare Level | 0.0262 | -0.0227 | -0.0221 | -0.0571 | -0.0996** |
|  | (0.413) | (-0.281) | (-0.267) | (-0.766) | (-2.505) |
| Ln(HIV) | 0.0477 | 0.0705* | 0.0861* | 0.106* | 0.129** |
|  | (1.161) | (1.727) | (1.826) | (1.840) | (2.470) |
| Region | Y | Y | Y | Y | Y |
| Observations | 80 | 92 | 92 | 92 | 90 |
| Adj. R-squared | 0.904 | 0.921 | 0.901 | 0.888 | 0.880 |
| F | . | . | . | . | . |
| Panel B: Dependent Variable: U5MR, female | | | | | |
| PMHGRP(=1) | 0.113 | 0.243*** | 0.193* | 0.147 | 0.0699 |
|  | (1.112) | (2.760) | (1.702) | (1.271) | (0.541) |
| Ln(Educ) | -0.949*** | -1.049*** | -0.842*** | -0.967*** | -0.991*** |
|  | (-2.815) | (-3.857) | (-2.948) | (-2.772) | (-2.967) |
| Ln(GDP per capita) | -0.482*** | -0.470*** | -0.456*** | -0.441*** | -0.353*** |
|  | (-7.518) | (-9.125) | (-7.423) | (-6.286) | (-5.111) |
| Ln(PM2.5) | 0.265** | 0.185* | 0.161 | 0.161 | 0.170* |
|  | (2.575) | (1.903) | (1.435) | (1.360) | (1.685) |
| SmokGRP | 0.0584 | 0.0703 | 0.156 | 0.0989 | -0.0301 |
|  | (0.573) | (0.811) | (1.515) | (1.071) | (-0.259) |
| AlcohGRP | 0.0968 | 0.135 | 0.104 | 0.0556 | 0.0161 |
|  | (0.945) | (1.552) | (1.005) | (0.384) | (0.135) |
| Ln(Basic Water) | -0.230 | 0.0877 | 0.224 | 0.173 | 0.189 |
|  | (-1.001) | (0.417) | (0.822) | (0.414) | (0.315) |
| Ln(Basic Sanitation) | -0.192** | -0.0864 | -0.165 | -0.209 | -0.100 |
|  | (-2.286) | (-1.271) | (-1.580) | (-1.302) | (-0.540) |
| Ln(Urban Pop.) | 0.274* | 0.146 | 0.114 | 0.216 | 0.238 |
|  | (1.893) | (1.026) | (0.679) | (1.222) | (1.135) |
| Healthcare Level | 0.0235 | -0.0226 | -0.0229 | -0.0604 | -0.100** |
|  | (0.376) | (-0.286) | (-0.281) | (-0.814) | (-2.543) |
| Ln(HIV) | 0.0504 | 0.0723* | 0.0880* | 0.107* | 0.127** |
|  | (1.198) | (1.736) | (1.846) | (1.812) | (2.441) |
| Region | Y | Y | Y | Y | Y |
| Observations | 80 | 92 | 92 | 92 | 90 |
| Adj. R-squared | 0.908 | 0.924 | 0.905 | 0.890 | 0.882 |
| F | . | . | . | . | . |
| Panel C: Dependent Variable: U5MR, male | | | | | |
| PMHGRP(=1) | 0.104 | 0.238*** | 0.190* | 0.155 | 0.0799 |
|  | (1.072) | (2.719) | (1.681) | (1.343) | (0.604) |
| Ln(Educ) | -0.850** | -0.920*** | -0.727** | -0.865** | -0.946*** |
|  | (-2.486) | (-3.374) | (-2.526) | (-2.522) | (-2.803) |
| Ln(GDP per capita) | -0.477*** | -0.476*** | -0.466*** | -0.451*** | -0.360*** |
|  | (-7.537) | (-9.131) | (-7.464) | (-6.409) | (-5.181) |
| Ln(PM2.5) | 0.240** | 0.170* | 0.150 | 0.155 | 0.165 |
|  | (2.342) | (1.724) | (1.328) | (1.308) | (1.626) |
| SmokGRP | 0.0548 | 0.0777 | 0.156 | 0.106 | -0.0415 |
|  | (0.555) | (0.920) | (1.547) | (1.143) | (-0.345) |
| AlcohGRP | 0.0942 | 0.126 | 0.0904 | 0.0461 | 0.0118 |
|  | (0.936) | (1.449) | (0.882) | (0.311) | (0.0972) |
| Ln(Basic Water) | -0.234 | 0.0811 | 0.212 | 0.175 | 0.257 |
|  | (-1.043) | (0.390) | (0.777) | (0.411) | (0.426) |
| Ln(Basic Sanitation) | -0.180** | -0.0784 | -0.159 | -0.203 | -0.112 |
|  | (-2.174) | (-1.181) | (-1.562) | (-1.281) | (-0.617) |
| Ln(Urban Pop.) | 0.264* | 0.134 | 0.103 | 0.196 | 0.204 |
|  | (1.849) | (0.939) | (0.617) | (1.100) | (0.962) |
| Healthcare Level | 0.0283 | -0.0223 | -0.0210 | -0.0562 | -0.0991** |
|  | (0.439) | (-0.273) | (-0.251) | (-0.753) | (-2.464) |
| Ln(HIV) | 0.0460 | 0.0695* | 0.0853* | 0.105* | 0.131** |
|  | (1.138) | (1.728) | (1.829) | (1.863) | (2.497) |
| Region | Y | Y | Y | Y | Y |
| Observations | 80 | 92 | 92 | 92 | 90 |
| Adj. R-squared | 0.901 | 0.919 | 0.899 | 0.887 | 0.877 |
| F | . | . | . | . | . |

Note: *** p<0.01, ** p<0.05, * p<0.1. Robust t-statistics in parentheses. Column (1) to Column (5) are the regression results from year 1990, 2000, 2005, 2010, 2020, respectively.

**Table A4 The regression results on determinants of U5MR in each time point, PMH in males**

|  | (1) | (2) | (3) | (4) | (5) |
| --- | --- | --- | --- | --- | --- |
| VARIABLES | 1990 | 2000 | 2005 | 2010 | 2019 |
| Panel A: Dependent Variable: U5MR | | | | | |
| PMHGRP(=1) | 0.121 | -0.0459 | -0.0896 | -0.0498 | -0.144 |
|  | (0.936) | (-0.316) | (-0.717) | (-0.372) | (-1.120) |
| Ln(Educ) | -0.762** | -0.762*** | -0.631** | -0.749** | -0.932*** |
|  | (-2.447) | (-2.982) | (-2.226) | (-2.154) | (-2.975) |
| Ln(GDP per capita) | -0.476*** | -0.469*** | -0.470*** | -0.454*** | -0.359*** |
|  | (-7.347) | (-8.772) | (-7.313) | (-6.083) | (-5.187) |
| Ln(PM2.5) | 0.235** | 0.205** | 0.196* | 0.170 | 0.183* |
|  | (2.205) | (2.041) | (1.774) | (1.429) | (1.817) |
| SmokGRP | 0.0745 | 0.132 | 0.185* | 0.0977 | -0.0516 |
|  | (0.771) | (1.488) | (1.743) | (1.013) | (-0.412) |
| AlcohGRP | 0.0551 | 0.133 | 0.144 | 0.0754 | 0.0571 |
|  | (0.554) | (1.427) | (1.385) | (0.476) | (0.481) |
| Ln(Basic Water) | -0.232 | 0.0284 | 0.184 | 0.197 | 0.338 |
|  | (-1.006) | (0.129) | (0.657) | (0.447) | (0.542) |
| Ln(Basic Sanitation) | -0.177** | -0.0901 | -0.180* | -0.239 | -0.133 |
|  | (-2.195) | (-1.262) | (-1.787) | (-1.460) | (-0.724) |
| Ln(Urban Pop.) | 0.264* | 0.187 | 0.182 | 0.214 | 0.222 |
|  | (1.842) | (1.316) | (1.157) | (1.211) | (1.041) |
| Healthcare Level | 0.0260 | -0.00293 | -0.0108 | -0.0461 | -0.108*** |
|  | (0.414) | (-0.0371) | (-0.134) | (-0.571) | (-2.699) |
| Ln(HIV) | 0.0503 | 0.102** | 0.113** | 0.129** | 0.149*** |
|  | (1.269) | (2.581) | (2.479) | (2.266) | (2.868) |
| Region | Y | Y | Y | Y | Y |
| Observations | 80 | 92 | 92 | 92 | 90 |
| Adj. R-squared | 0.905 | 0.915 | 0.898 | 0.885 | 0.881 |
| F | . | . | . | . | . |
| Panel B: Dependent Variable: U5MR, female | | | | | |
| PMHGRP(=1) | 0.132 | -0.0445 | -0.0822 | -0.0503 | -0.137 |
|  | (1.018) | (-0.309) | (-0.664) | (-0.378) | (-1.075) |
| Ln(Educ) | -0.810** | -0.831*** | -0.693** | -0.812** | -0.960*** |
|  | (-2.631) | (-3.261) | (-2.458) | (-2.302) | (-3.072) |
| Ln(GDP per capita) | -0.480*** | -0.466*** | -0.464*** | -0.449*** | -0.355*** |
|  | (-7.296) | (-8.753) | (-7.274) | (-6.060) | (-5.113) |
| Ln(PM2.5) | 0.247** | 0.214** | 0.202* | 0.174 | 0.185* |
|  | (2.317) | (2.129) | (1.833) | (1.462) | (1.843) |
| SmokGRP | 0.0778 | 0.129 | 0.185* | 0.0936 | -0.0446 |
|  | (0.793) | (1.428) | (1.735) | (0.976) | (-0.361) |
| AlcohGRP | 0.0539 | 0.138 | 0.150 | 0.0807 | 0.0571 |
|  | (0.535) | (1.467) | (1.438) | (0.518) | (0.486) |
| Ln(Basic Water) | -0.230 | 0.0313 | 0.187 | 0.197 | 0.295 |
|  | (-0.984) | (0.141) | (0.671) | (0.454) | (0.475) |
| Ln(Basic Sanitation) | -0.183** | -0.0945 | -0.181* | -0.241 | -0.126 |
|  | (-2.252) | (-1.307) | (-1.785) | (-1.466) | (-0.677) |
| Ln(Urban Pop.) | 0.269* | 0.194 | 0.187 | 0.227 | 0.239 |
|  | (1.866) | (1.367) | (1.197) | (1.288) | (1.133) |
| Healthcare Level | 0.0230 | -0.00256 | -0.0111 | -0.0501 | -0.108*** |
|  | (0.372) | (-0.0332) | (-0.140) | (-0.625) | (-2.733) |
| Ln(HIV) | 0.0529 | 0.104** | 0.114** | 0.129** | 0.146*** |
|  | (1.316) | (2.587) | (2.484) | (2.217) | (2.817) |
| Region | Y | Y | Y | Y | Y |
| Observations | 80 | 92 | 92 | 92 | 90 |
| Adj. R-squared | 0.908 | 0.918 | 0.902 | 0.888 | 0.883 |
| F | . | . | . | . | . |
| Panel C: Dependent Variable: U5MR, male | | | | | |
| PMHGRP(=1) | 0.116 | -0.0492 | -0.0928 | -0.0509 | -0.151 |
|  | (0.894) | (-0.335) | (-0.741) | (-0.378) | (-1.159) |
| Ln(Educ) | -0.724** | -0.709*** | -0.584** | -0.701** | -0.910*** |
|  | (-2.294) | (-2.768) | (-2.069) | (-2.046) | (-2.889) |
| Ln(GDP per capita) | -0.474*** | -0.472*** | -0.474*** | -0.459*** | -0.362*** |
|  | (-7.366) | (-8.788) | (-7.353) | (-6.162) | (-5.181) |
| Ln(PM2.5) | 0.224** | 0.199* | 0.191* | 0.168 | 0.182* |
|  | (2.098) | (1.965) | (1.735) | (1.407) | (1.806) |
| SmokGRP | 0.0728 | 0.135 | 0.186* | 0.100 | -0.0574 |
|  | (0.760) | (1.537) | (1.756) | (1.039) | (-0.449) |
| AlcohGRP | 0.0558 | 0.129 | 0.139 | 0.0720 | 0.0573 |
|  | (0.565) | (1.385) | (1.337) | (0.449) | (0.480) |
| Ln(Basic Water) | -0.235 | 0.0262 | 0.178 | 0.199 | 0.374 |
|  | (-1.025) | (0.120) | (0.637) | (0.449) | (0.598) |
| Ln(Basic Sanitation) | -0.172** | -0.0866 | -0.177* | -0.236 | -0.140 |
|  | (-2.135) | (-1.225) | (-1.776) | (-1.451) | (-0.771) |
| Ln(Urban Pop.) | 0.260* | 0.182 | 0.179 | 0.207 | 0.206 |
|  | (1.816) | (1.278) | (1.142) | (1.169) | (0.963) |
| Healthcare Level | 0.0281 | -0.00290 | -0.0102 | -0.0452 | -0.108*** |
|  | (0.442) | (-0.0361) | (-0.126) | (-0.559) | (-2.657) |
| Ln(HIV) | 0.0487 | 0.101** | 0.112** | 0.128** | 0.152*** |
|  | (1.238) | (2.586) | (2.482) | (2.303) | (2.916) |
| Region | Y | Y | Y | Y | Y |
| Observations | 80 | 92 | 92 | 92 | 90 |
| Adj. R-squared | 0.902 | 0.913 | 0.896 | 0.884 | 0.878 |
| F | . | . | . | . | . |

Note: *** p<0.01, ** p<0.05, * p<0.1. Robust t-statistics in parentheses. Column (1) to Column (5) are the regression results from year 1990, 2000, 2005, 2010, 2020, respectively.

**Table A5 List of countries included in regression analysis**

| Albania | Haiti | Panama |
| --- | --- | --- |
| Algeria | Iceland | Paraguay |
| Argentina | Indonesia | Peru |
| Armenia | Iran, Islamic Rep. | Philippines |
| Australia | Ireland | Portugal |
| Benin | Italy | Qatar |
| Botswana | Jamaica | Romania |
| Brazil | Japan | Rwanda |
| Burkina Faso | Kazakhstan | Saudi Arabia |
| Burundi | Kenya | Senegal |
| Cambodia | Kyrgyz Republic | Serbia |
| Cameroon | Lao PDR | Sierra Leone |
| Chile | Lesotho | Singapore |
| Colombia | Liberia | Slovenia |
| Congo, Rep. | Madagascar | South Africa |
| Costa Rica | Malawi | Spain |
| Cote d'Ivoire | Malaysia | Sri Lanka |
| Croatia | Mali | Switzerland |
| Denmark | Mauritius | Tanzania |
| Dominican Republic | Mexico | Thailand |
| Egypt, Arab Rep. | Moldova | Togo |
| El Salvador | Mongolia | Tunisia |
| Estonia | Morocco | Uganda |
| Eswatini | Mozambique | Ukraine |
| Ethiopia | Namibia | United Arab Emirates |
| Fiji | Nepal | United States |
| Gambia, The | Netherlands | Uruguay |
| Germany | New Zealand | Yemen, Rep. |
| Ghana | Niger | Zambia |
| Greece | Nigeria | Zimbabwe |
| Guyana | Pakistan |  |

**Table A6-1** The ratio of average U5MR between countries in high PMH group and those with low PMH group, PMH in females

|  | | Data | Data | Data | Data | Change in data |  |
| --- | --- | --- | --- | --- | --- | --- | --- |
|  | | 1990 | 2000 | 2010 | 2019 | $\Delta_{1990-2019}$ | |
| Correlation 1990 | | **1.0369** | 1.0290 | 1.0331 | 1.0328 | -0.0042 | |
|  |  | **(0.0006)** | (0.0005) | (0.0005) | (0.0006) | (0.0008) | |
| Correlation 2000 | | 1.0935 | **1.0747** | 1.0834 | 1.0824 | -0.0111 | |
|  |  | (0.0014) | **(0.0012)** | (0.0012) | (0.0014) | (0.0020) | |
| Correlation 2010 | | 1.0562 | 1.0450 | **1.0508** | 1.0504 | -0.0058 | |
|  |  | (0.0009) | (0.0007) | **(0.0008)** | (0.0009) | (0.0013) | |
| Correlation 2019 | | 1.0279 | 1.0221 | 1.0247 | **1.0243** | -0.0036 | |
|  |  | (0.0004) | (0.0003) | (0.0004) | **(0.0004)** | (0.0006) | |
| Change in  correlation | $\Delta_{1990-2019}$ | -0.0090 | -0.0069 | -0.0084 | -0.0084 | -0.0126 | |
|  |  | (0.0002) | (0.0002) | (0.0002) | (0.0002) | (0.0007) | |

Note: The under-five mortality ratio is calculated as $\hat{r}={\bar{\hat{U5MR}}}_{PMHGRP=1}/{\bar{\hat{U5MR}}}_{PMHGRP=0}$, where $\bar{\hat{U5MR}}$stands for the mean of estimated U5MR. And the baseline prediction of $\hat{U5MR}$ uses PMH status, education, environment, GDP per capita, health behavior, water and sanitation, urbanization, healthcare level, and HIV prevalence. The reported ratios in table are computed as the mean of $\hat{r}$ across 200 bootstrap replications, standard errors in parentheses. In each bootstrap replication, we randomly draw 70% of the total sample.

**Table A6-2** The ratio of average U5MR (female) between countries in high PMH group and those with low PMH group, PMH in females

|  | | Data | Data | Data | Data | Change in data |  |
| --- | --- | --- | --- | --- | --- | --- | --- |
|  | | 1990 | 2000 | 2010 | 2019 | $\Delta_{1990-2019}$ | |
| Correlation 1990 | | **1.0381** | 1.0299 | 1.0342 | 1.0339 | -0.0042 | |
|  |  | **(0.0006)** | (0.0005) | (0.0005) | (0.0006) | (0.0009) | |
| Correlation 2000 | | 1.0930 | **1.0742** | 1.0830 | 1.0821 | -0.0108 | |
|  |  | (0.0014) | **(0.0012)** | (0.0013) | (0.0014) | (0.0020) | |
| Correlation 2010 | | 1.0532 | 1.0425 | **1.0480** | 1.0477 | -0.0055 | |
|  |  | (0.0009) | (0.0007) | **(0.0007)** | (0.0009) | (0.0012) | |
| Correlation 2019 | | 1.0266 | 1.0211 | 1.0235 | **1.0232** | -0.0034 | |
|  |  | (0.0004) | (0.0003) | (0.0003) | **(0.0004)** | (0.0006) | |
| Change in  correlation | $\Delta_{1990-2019}$ | -0.0115 | -0.0088 | -0.0108 | -0.0107 | **-0.0149** | |
|  |  | (0.0003) | (0.0002) | (0.0002) | (0.0002) | **(0.0008)** | |

Note: The under-five mortality ratio is calculated as $\hat{r}={\bar{\hat{U5MR}}}_{PMHGRP=1}/{\bar{\hat{U5MR}}}_{PMHGRP=0}$, where $\bar{\hat{U5MR}}$stands for the mean of estimated U5MR. And the baseline prediction of $\hat{U5MR}$ uses PMH status, education, environment, GDP per capita, health behavior, water and sanitation, urbanization, healthcare level, and HIV prevalence. The reported ratios in table are computed as the mean of $\hat{r}$ across 200 bootstrap replications, standard errors in parentheses. In each bootstrap replication, we randomly draw 70% of the total sample.

**Table A6-3** The ratio of average U5MR (male) between countries in high PMH group and those with low PMH group, PMH in females

|  | | Data | Data | Data | Data | Change in data |  |
| --- | --- | --- | --- | --- | --- | --- | --- |
|  | | 1990 | 2000 | 2010 | 2019 | $\Delta_{1990-2019}$ | |
| Correlation 1990 | | **1.0365** | 1.0287 | 1.0326 | 1.0324 | -0.0042 | |
|  |  | **(0.0006)** | (0.0005) | (0.0005) | (0.0006) | (0.0008) | |
| Correlation 2000 | | 1.0938 | **1.0752** | 1.0836 | 1.0826 | -0.0113 | |
|  |  | (0.0014) | **(0.0012)** | (0.0012) | (0.0014) | (0.0020) | |
| Correlation 2010 | | 1.0573 | 1.0459 | **1.0517** | 1.0513 | -0.0060 | |
|  |  | (0.0009) | (0.0008) | **(0.0008)** | (0.0009) | (0.0013) | |
| Correlation 2019 | | 1.0306 | 1.0243 | 1.0271 | **1.0267** | -0.0039 | |
|  |  | (0.0005) | (0.0004) | (0.0004) | **(0.0005)** | (0.0006) | |
| Change in  correlation | $\Delta_{1990-2019}$ | -0.0059 | -0.0044 | -0.0056 | -0.0057 | **-0.0098** | |
|  |  | (0.0002) | (0.0002) | (0.0001) | (0.0001) | **(0.0007)** | |

Note: The under-five mortality ratio is calculated as $\hat{r}={\bar{\hat{U5MR}}}_{PMHGRP=1}/{\bar{\hat{U5MR}}}_{PMHGRP=0}$, where $\bar{\hat{U5MR}}$stands for the mean of estimated U5MR. And the baseline prediction of $\hat{U5MR}$ uses PMH status, education, environment, GDP per capita, health behavior, water and sanitation, urbanization, healthcare level, and HIV prevalence. The reported ratios in table are computed as the mean of $\hat{r}$ across 200 bootstrap replications, standard errors in parentheses. In each bootstrap replication, we randomly draw 70% of the total sample.

**Table A7-1** The ratio of average U5MR between countries in high PMH group and those with low PMH group, PMH in males

|  | | Data | Data | Data | Data | Change in data |  |
| --- | --- | --- | --- | --- | --- | --- | --- |
|  | | 1990 | 2000 | 2010 | 2019 | $\Delta_{1990-2019}$ | |
| Correlation 1990 | | **1.0936** | 1.0994 | 1.0980 | 1.0939 | 0.0003 | |
|  |  | **(0.0006)** | (0.0005) | (0.0005) | (0.0007) | (0.0009) | |
| Correlation 2000 | | 0.9681 | **0.9659** | 0.9662 | 0.9671 | -0.0010 | |
|  |  | (0.0002) | **(0.0002)** | (0.0002) | (0.0002) | (0.0003) | |
| Correlation 2010 | | 0.9640 | 0.9616 | **0.9618** | 0.9630 | -0.0010 | |
|  |  | (0.0002) | (0.0002) | **(0.0002)** | (0.0002) | (0.0003) | |
| Correlation 2019 | | 0.9042 | 0.8963 | 0.8965 | **0.8976** | -0.0066 | |
|  |  | (0.0006) | (0.0005) | (0.0005) | **(0.0006)** | (0.0009) | |
| Change in  correlation | $\Delta_{1990-2019}$ | -0.0059 | -0.0044 | -0.0056 | -0.0057 | **-0.1960** | |
|  |  | (0.0002) | (0.0002) | (0.0001) | (0.0001) | **(0.0008)** | |

Note: The under-five mortality ratio is calculated as $\hat{r}={\bar{\hat{U5MR}}}_{PMHGRP=1}/{\bar{\hat{U5MR}}}_{PMHGRP=0}$, where $\bar{\hat{U5MR}}$stands for the mean of estimated U5MR. And the baseline prediction of $\hat{U5MR}$ uses PMH status, education, environment, GDP per capita, health behavior, water and sanitation, urbanization, healthcare level, and HIV prevalence. The reported ratios in table are computed as the mean of $\hat{r}$ across 200 bootstrap replications, standard errors in parentheses. In each bootstrap replication, we randomly draw 70% of the total sample.

**Table A7-2** The ratio of average U5MR (female) between countries in high PMH group and those with low PMH group, PMH in males

|  | | Data | Data | Data | Data | Change in data |  |
| --- | --- | --- | --- | --- | --- | --- | --- |
|  | | 1990 | 2000 | 2010 | 2019 | $\Delta_{1990-2019}$ | |
| Correlation 1990 | | **1.1030** | 1.1092 | 1.1078 | 1.1032 | 0.0002 | |
|  |  | **(0.0006)** | (0.0005) | (0.0006) | (0.0007) | (0.0010) | |
| Correlation 2000 | | 0.9689 | **0.9667** | 0.9670 | 0.9679 | -0.0010 | |
|  |  | (0.0002) | **(0.0002)** | (0.0002) | (0.0002) | (0.0003) | |
| Correlation 2010 | | 0.9635 | 0.9611 | **0.9614** | 0.9625 | -0.0010 | |
|  |  | (0.0002) | (0.0002) | **(0.0002)** | (0.0002) | (0.0003) | |
| Correlation 2019 | | 0.9082 | 0.9007 | 0.9009 | **0.9020** | -0.0062 | |
|  |  | (0.0006) | (0.0005) | (0.0005) | **(0.0006)** | (0.0008) | |
| Change in  correlation | $\Delta_{1990-2019}$ | -0.1948 | -0.2084 | -0.2068 | -0.2012 | **-0.2010** | |
|  |  | (0.0012) | (0.0010) | (0.0011) | (0.0013) | **(0.0008)** | |

Note: The under-five mortality ratio is calculated as $\hat{r}={\bar{\hat{U5MR}}}_{PMHGRP=1}/{\bar{\hat{U5MR}}}_{PMHGRP=0}$, where $\bar{\hat{U5MR}}$stands for the mean of estimated U5MR. And the baseline prediction of $\hat{U5MR}$ uses PMH status, education, environment, GDP per capita, health behavior, water and sanitation, urbanization, healthcare level, and HIV prevalence. The reported ratios in table are computed as the mean of $\hat{r}$ across 200 bootstrap replications, standard errors in parentheses. In each bootstrap replication, we randomly draw 70% of the total sample.

**Table A7-3** The ratio of average U5MR (male) between countries in high PMH group and those with low PMH group, PMH in males

|  | | Data | Data | Data | Data | Change in data |  |
| --- | --- | --- | --- | --- | --- | --- | --- |
|  | | 1990 | 2000 | 2010 | 2019 | $\Delta_{1990-2019}$ | |
| Correlation 1990 | | **1.0888** | 1.0944 | 1.0931 | 1.0891 | 0.0003 | |
|  |  | **(0.0005)** | (0.0005) | (0.0005) | (0.0006) | (0.0009) | |
| Correlation 2000 | | 0.9661 | **0.9636** | 0.9639 | 0.9649 | -0.0012 | |
|  |  | (0.0002) | **(0.0002)** | (0.0002) | (0.0002) | (0.0003) | |
| Correlation 2010 | | 0.9633 | 0.9608 | **0.9611** | 0.9622 | -0.0011 | |
|  |  | (0.0002) | (0.0002) | **(0.0002)** | (0.0002) | (0.0003) | |
| Correlation 2019 | | 0.8995 | 0.8911 | 0.8913 | **0.8924** | -0.0071 | |
|  |  | (0.0006) | (0.0005) | (0.0005) | **(0.0006)** | (0.0009) | |
| Change in  correlation | $\Delta_{1990-2019}$ | -0.1893 | -0.2032 | -0.2018 | -0.1967 | **-0.1964** | |
|  |  | (0.0011) | (0.0010) | (0.0010) | (0.0013) | **(0.0008)** | |

Note: The under-five mortality ratio is calculated as $\hat{r}={\bar{\hat{U5MR}}}_{PMHGRP=1}/{\bar{\hat{U5MR}}}_{PMHGRP=0}$, where $\bar{\hat{U5MR}}$stands for the mean of estimated U5MR. And the baseline prediction of $\hat{U5MR}$ uses PMH status, education, environment, GDP per capita, health behavior, water and sanitation, urbanization, healthcare level, and HIV prevalence. The reported ratios in table are computed as the mean of $\hat{r}$ across 200 bootstrap replications, standard errors in parentheses. In each bootstrap replication, we randomly draw 70% of the total sample.

**Table A8** **The regression results on determinants of U5MR in each time point, by using DALYs as the grouping variable**

|  | (1) | (2) | (3) | (4) | (5) |
| --- | --- | --- | --- | --- | --- |
|  | 1990 | 2000 | 2005 | 2010 | 2019 |
| VARIABLES | Ln(U5MR) | Ln(U5MR) | Ln(U5MR) | Ln(U5MR) | Ln(U5MR) |
| PMHGRP(=1) | 0.00922 | -0.199* | -0.203* | -0.179* | -0.191** |
|  | (0.0796) | (-1.985) | (-1.873) | (-1.723) | (-1.999) |
| Ln(Educ) | -0.810** | -0.838*** | -0.701** | -0.847** | -1.084*** |
|  | (-2.384) | (-3.299) | (-2.551) | (-2.640) | (-3.531) |
| Ln(GDP per capita) | -0.476*** | -0.437*** | -0.444*** | -0.436*** | -0.334*** |
|  | (-6.868) | (-8.518) | (-7.068) | (-5.794) | (-4.766) |
| Ln(PM2.5) | 0.258** | 0.158 | 0.125 | 0.108 | 0.142 |
|  | (2.525) | (1.624) | (1.136) | (0.897) | (1.465) |
| SmokGRP | 0.0774 | 0.126 | 0.178* | 0.114 | -0.0343 |
|  | (0.784) | (1.443) | (1.744) | (1.199) | (-0.297) |
| AlcohGRP | 0.0816 | 0.155* | 0.173 | 0.0872 | 0.0812 |
|  | (0.838) | (1.716) | (1.562) | (0.614) | (0.666) |
| Ln(Basic Water) | -0.241 | -0.0675 | 0.0794 | 0.0667 | 0.190 |
|  | (-1.032) | (-0.320) | (0.284) | (0.151) | (0.320) |
| Ln(Basic Sanitation) | -0.179** | -0.105* | -0.199** | -0.263* | -0.153 |
|  | (-2.202) | (-1.717) | (-2.073) | (-1.849) | (-0.892) |
| Ln(Urban Pop.) | 0.292* | 0.135 | 0.126 | 0.199 | 0.214 |
|  | (1.996) | (0.966) | (0.785) | (1.124) | (1.008) |
| Healthcare Level | 0.0312 | 0.0394 | 0.0301 | -0.00915 | -0.0859** |
|  | (0.466) | (0.479) | (0.349) | (-0.110) | (-1.994) |
| Ln(HIV) | 0.0584 | 0.103*** | 0.114** | 0.132** | 0.143*** |
|  | (1.403) | (2.733) | (2.470) | (2.387) | (2.741) |
| Region | Y | Y | Y | Y | Y |
| Observations | 80 | 92 | 92 | 92 | 90 |
| Adj. R-squared | 0.903 | 0.920 | 0.902 | 0.889 | 0.884 |
| F | NA | NA | NA | NA | NA |

Note: *** p<0.01, ** p<0.05, * p<0.1. Robust t-statistics in parentheses. Column (1) to Column (5) are the regression results from year 1990, 2000, 2010, 2019, respectively. PMH was measured by DALYs per 100,000 population due to population mental disorders.


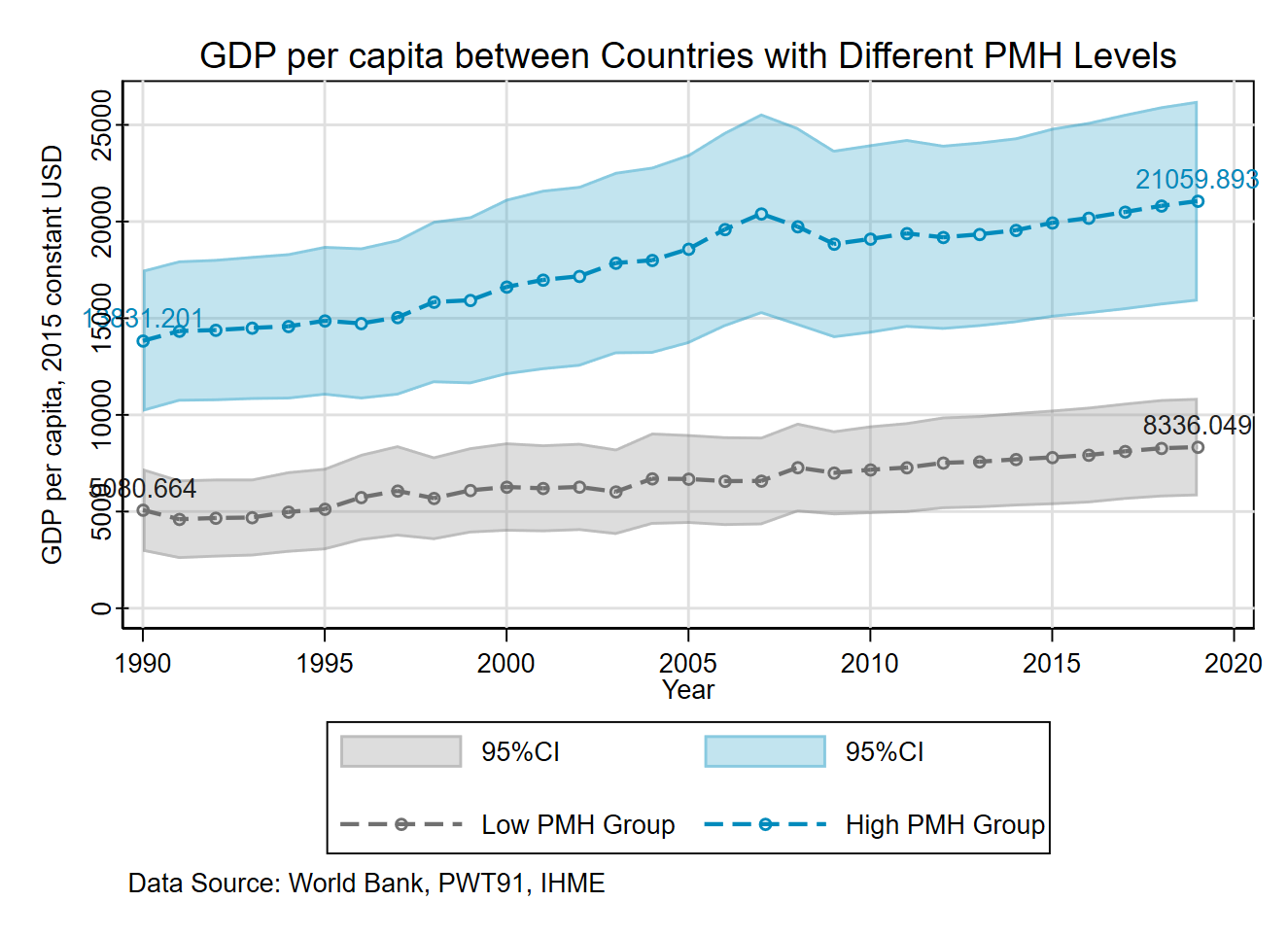


**Figure A1-a GDP per capita, by PMH Group**


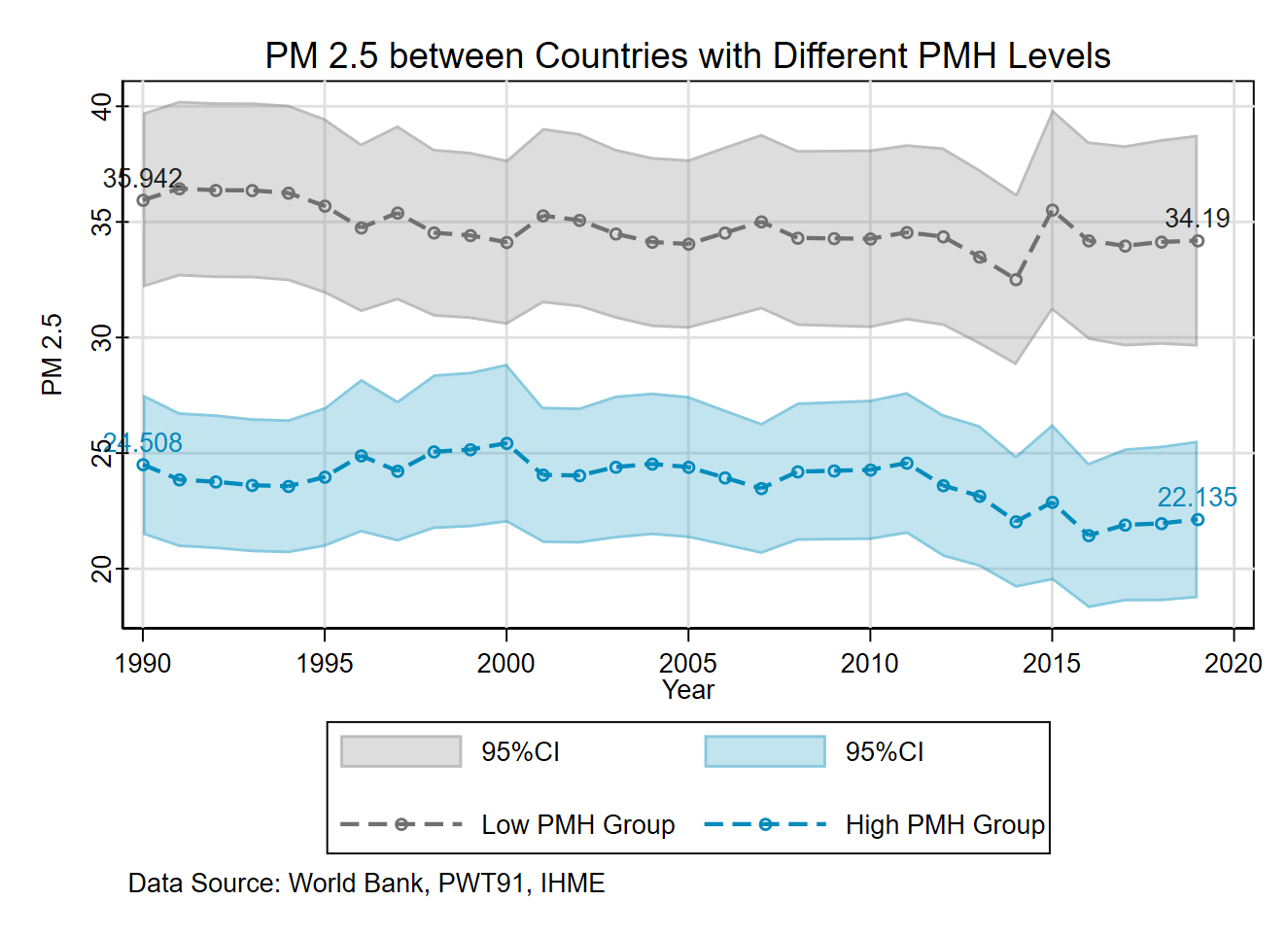


**Figure A1-b PM2.5, by PMH Group**


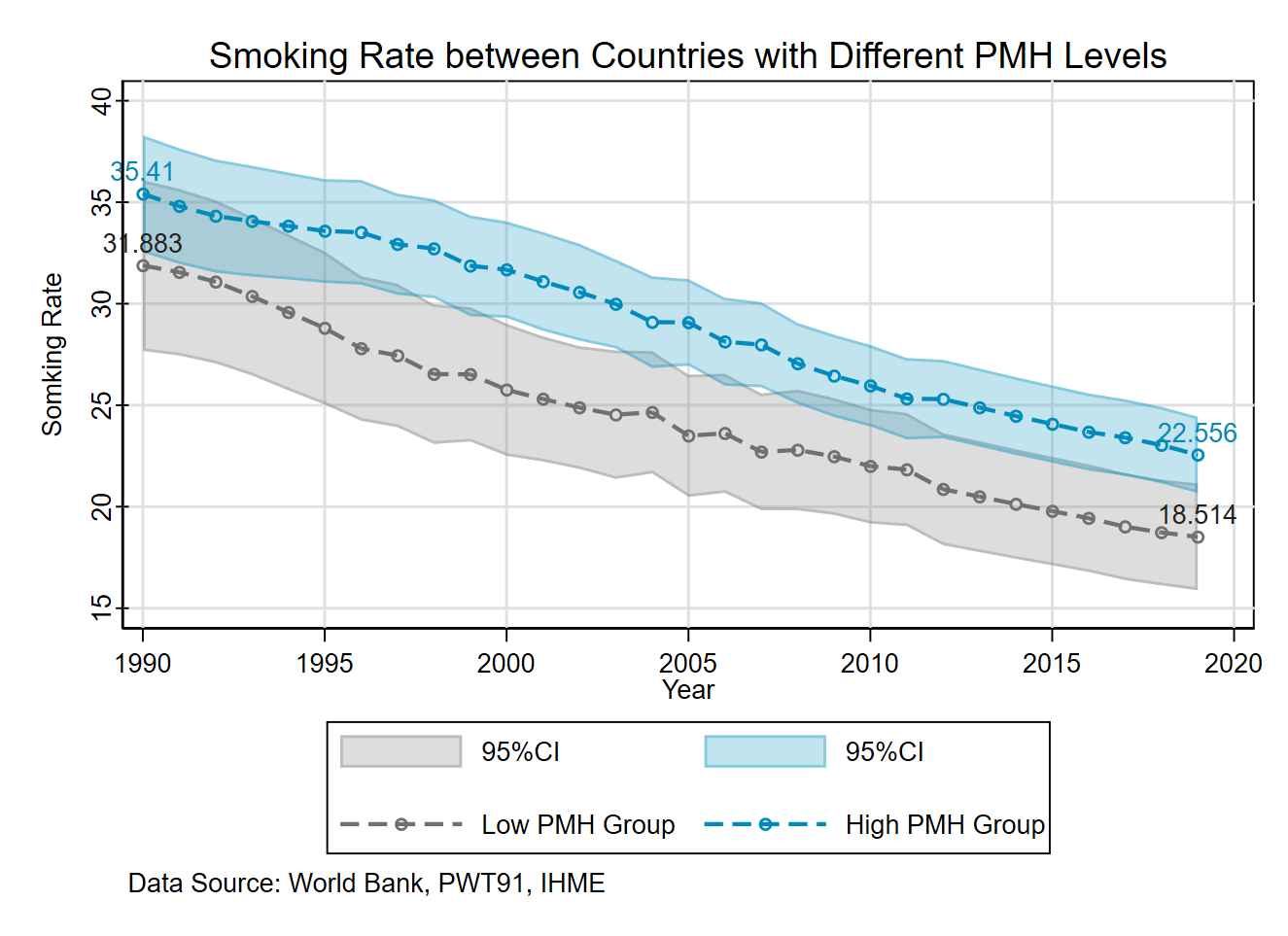


**Figure A1-c Smoking Rate, by PMH Group**


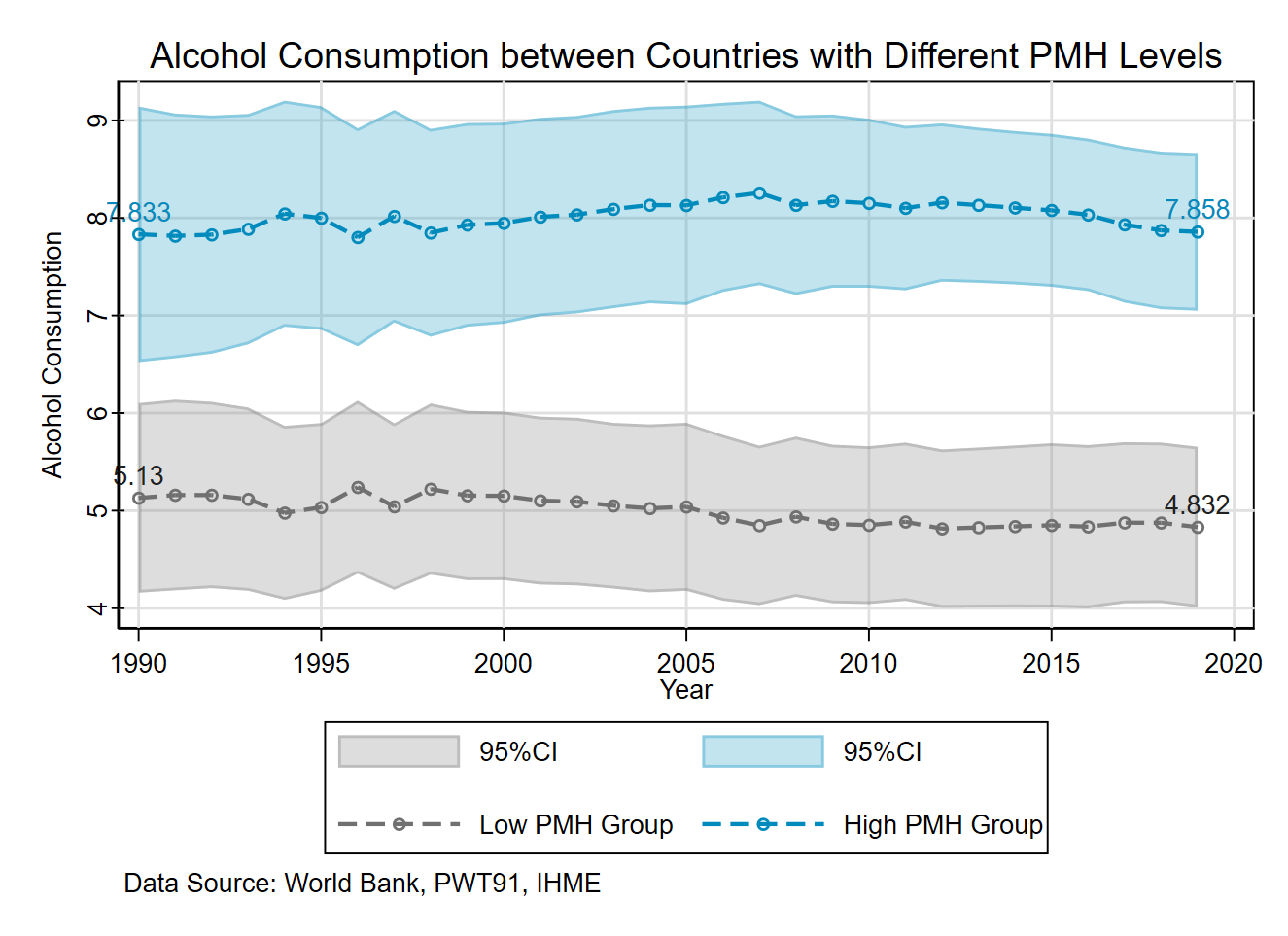


**Figure A1-d Alcohol Consumption, by PMH Group**

**Figure A1 PMH and Control Variables**


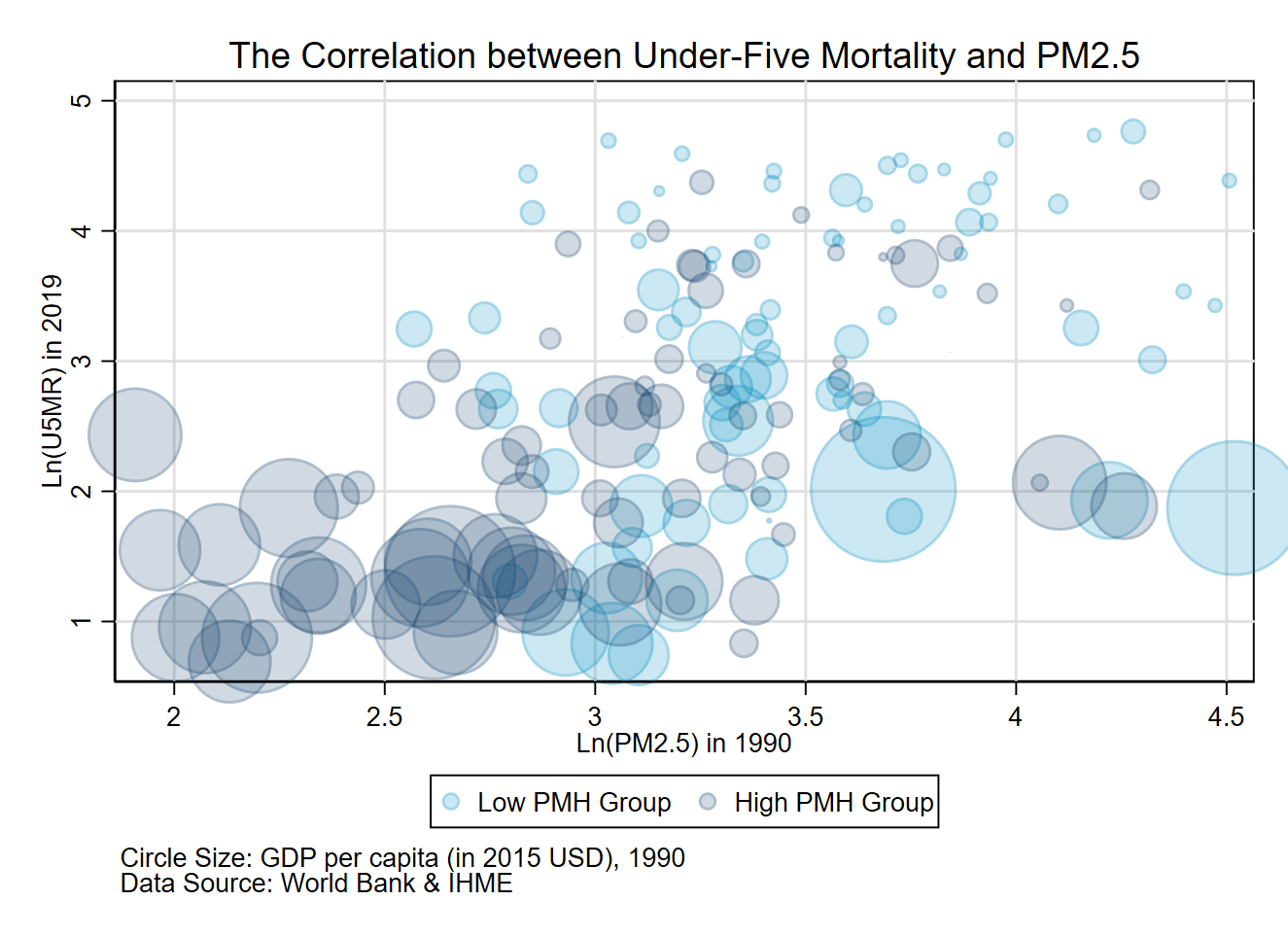


**Figure A2 the Correlation between Under-Five Mortality and PM2.5, by PMH Group**


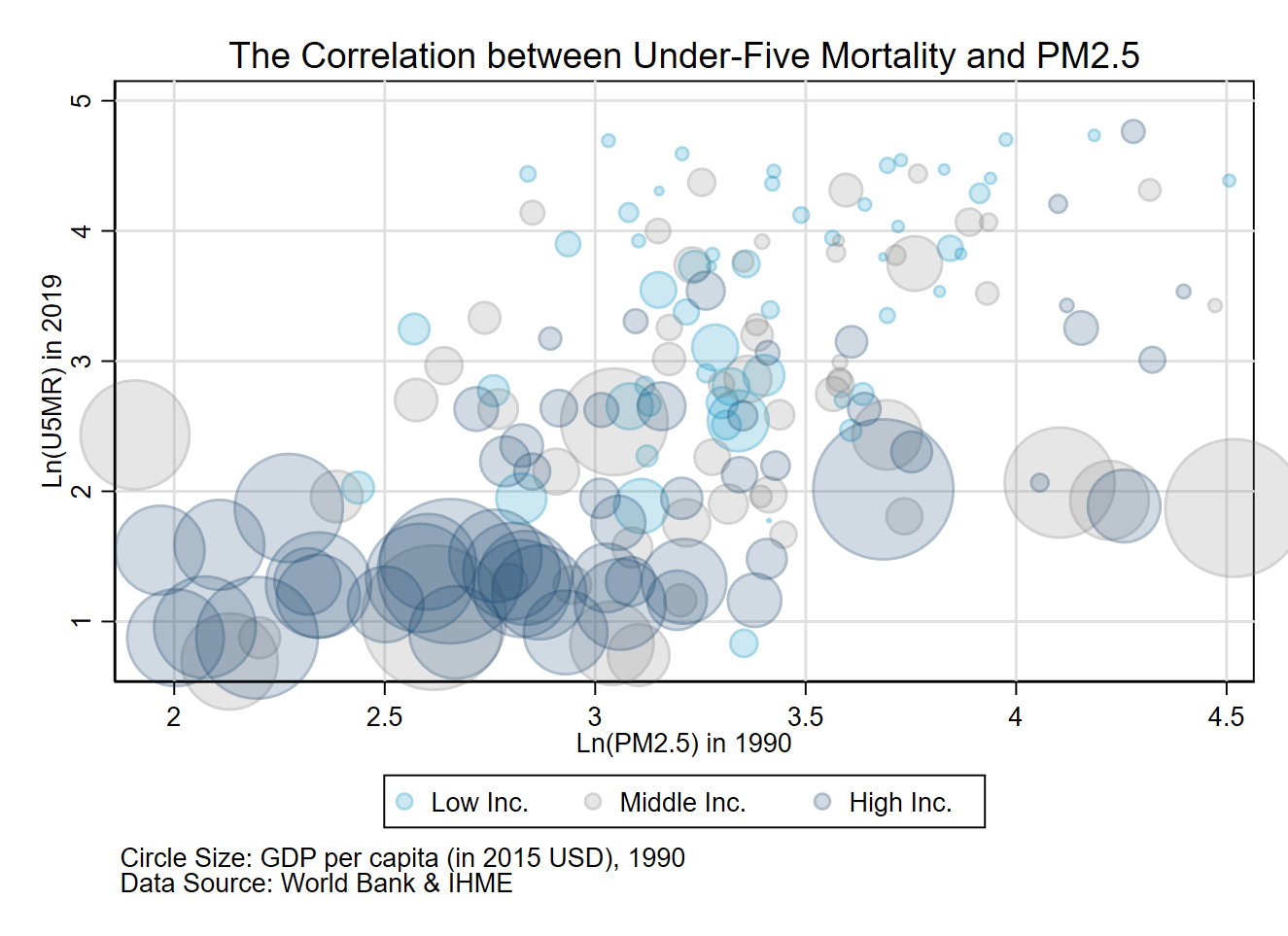


**Figure A3 the Correlation between Under-Five Mortality and PM2.5, by Economic Status**


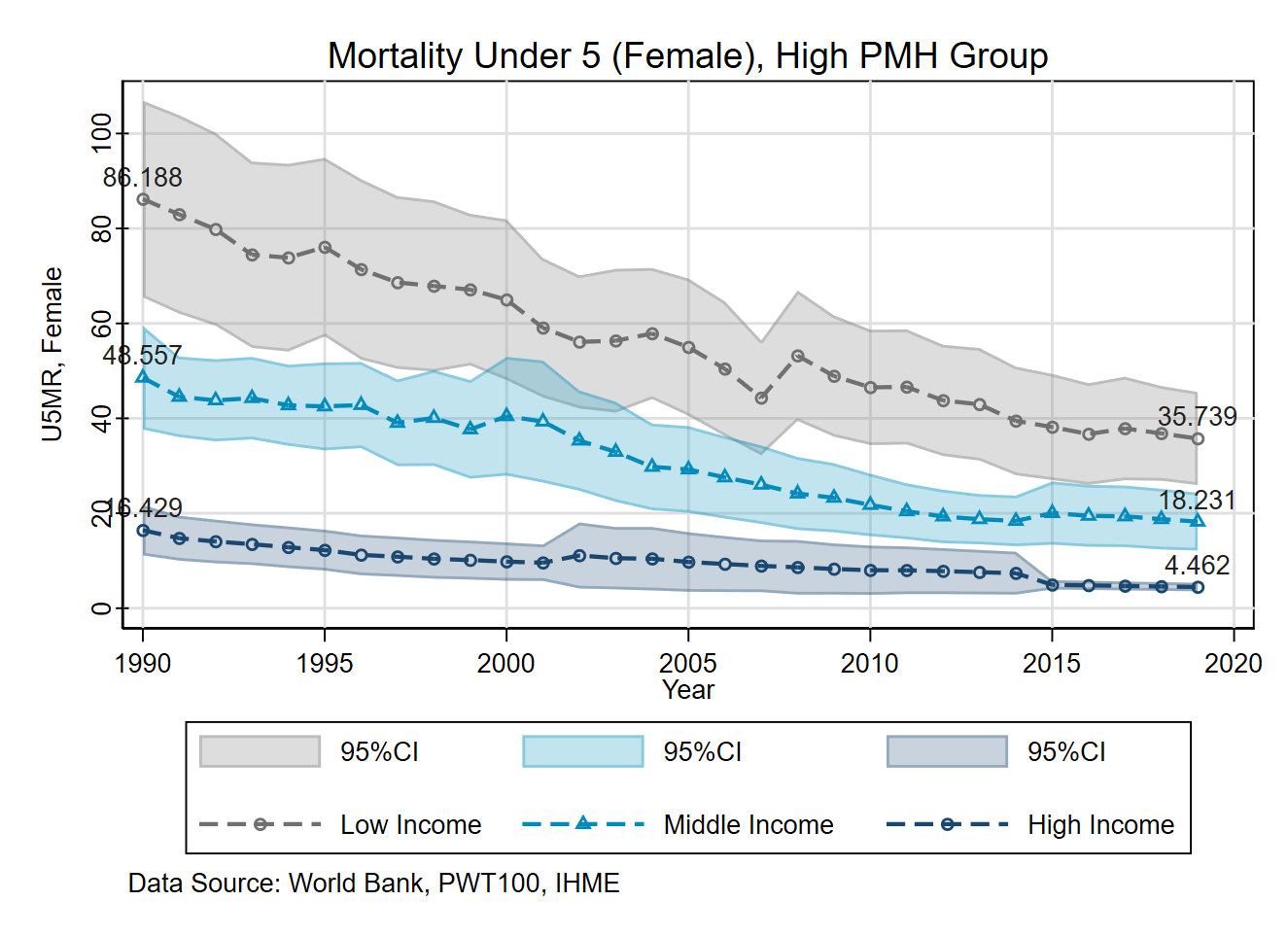


**Figure A4-a High PMH Group**


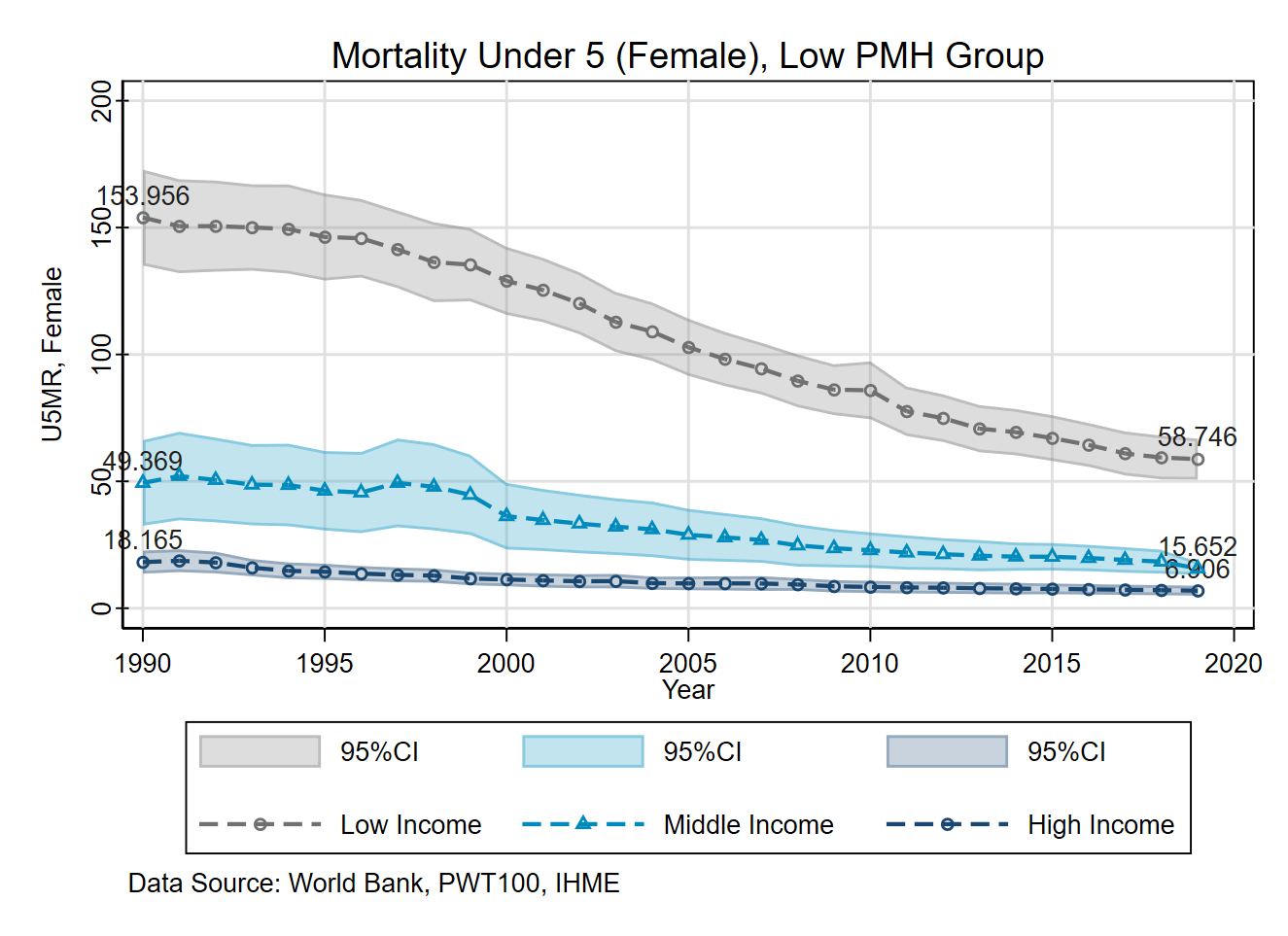


**Figure A4-b Low PMH Group**

**Figure A4 PMH-Mortality (Female) Gradient and Income**


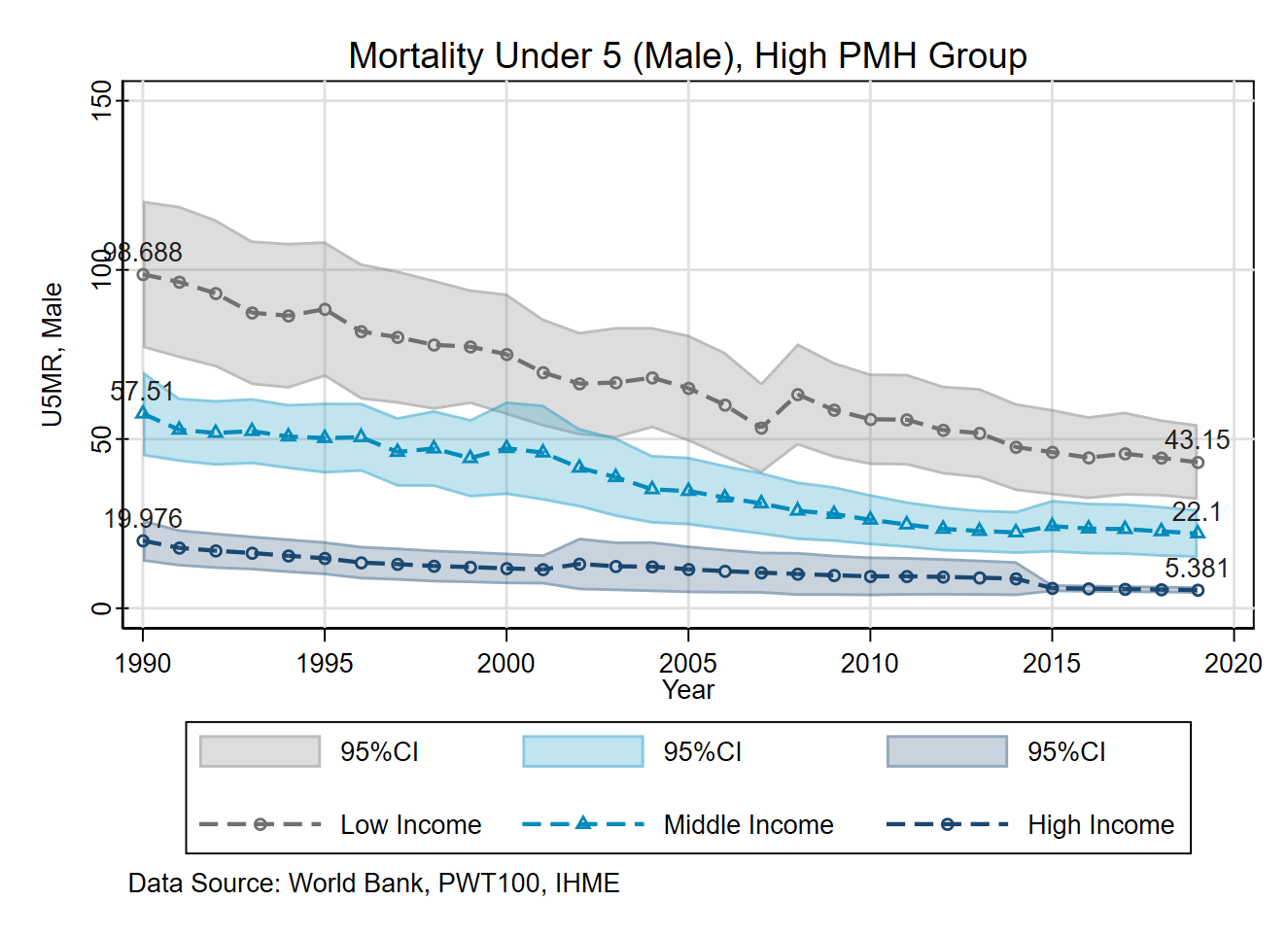


**Figure A5-a High PMH Group**


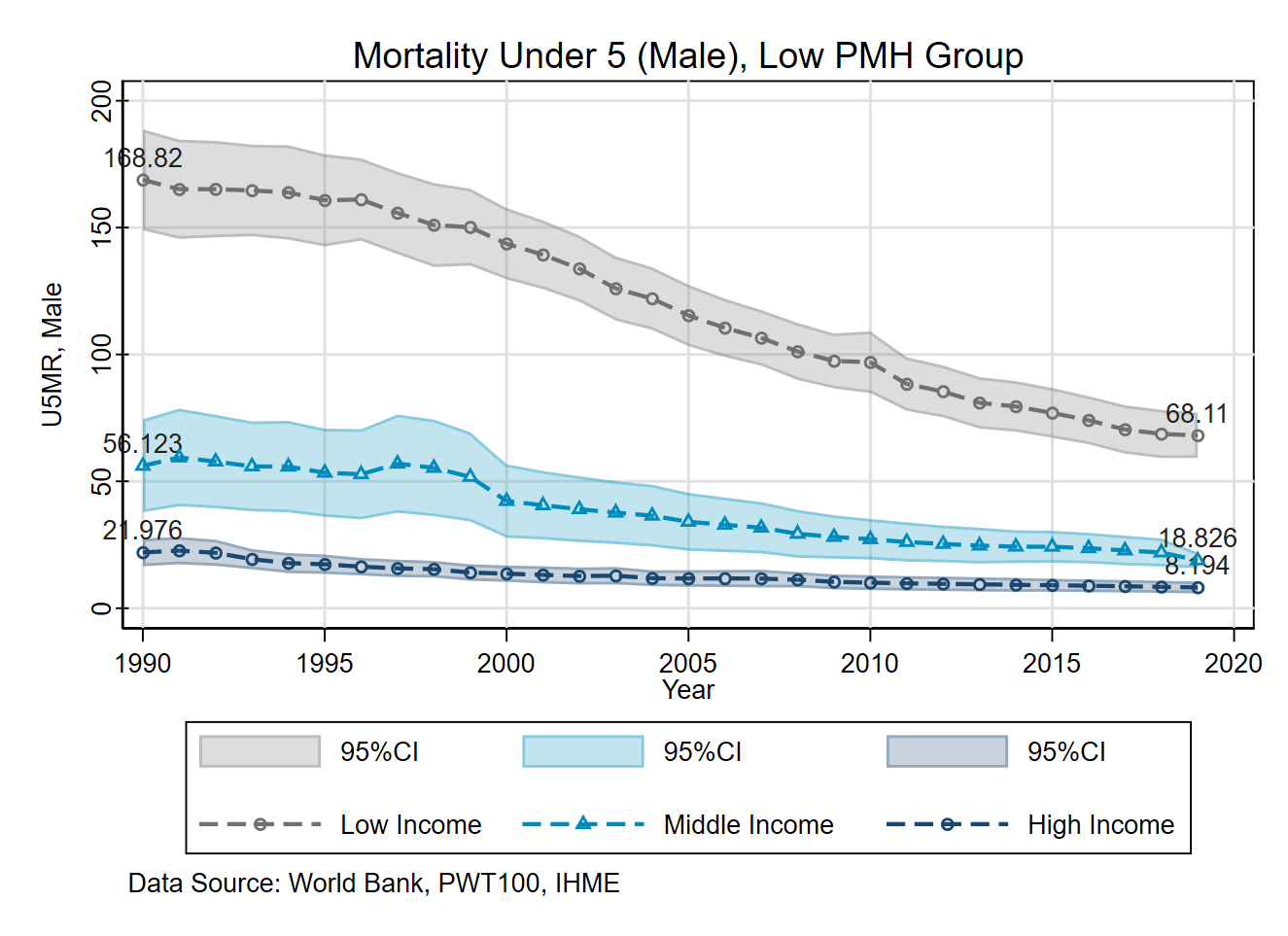


**Figure A5-b Low PMH Group**

**Figure A5 PMH-Mortality (Male) Gradient and Income**


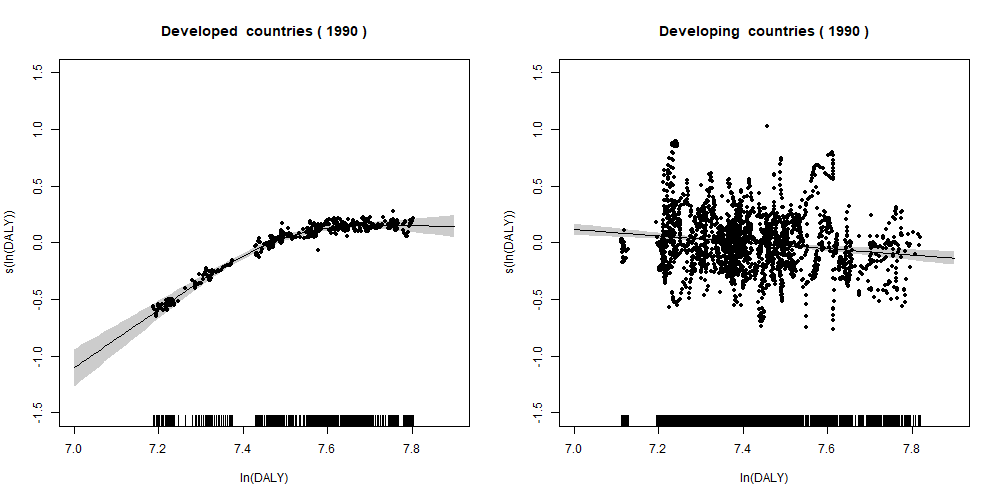


**FigureA6-1 DALYs and U5MR, grouping based on 1990 World Bank standard**

Note: The horizontal axis is DALY in log, and the vertical axis is the fitted U5MR that can be explained by DALYs. We divide sample into developed (left panel) and developing (right panel) group based on the World Bank standard. It is clear that, for the developed countries, as the DALYs goes up, the U5MR increase, however for the developing countries, there is a slightly inverse relationship between DALYs and U5MR.


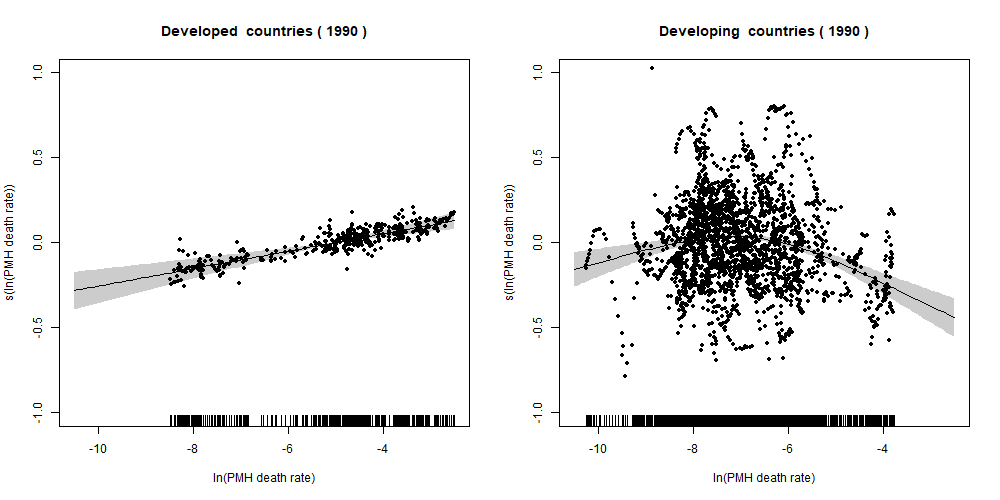


**Figure A6-2 Death Rate and U5MR, grouping based on 1990 World Bank standard**

Note: The horizontal axis is Death Rate in natural log form, and the vertical axis is the fitted U5MR that can be explained by Death Rate. We divide sample into developed (left panel) and developing (right panel) group based on the World Bank standard. It is clear that, for both of the developed and developing countries, as the Death Rate goes up, the U5MR decrease.

**Figure A6 Mental Disorder DALYs, Death Rate and U5MR, GAM**
